# Literacy-related differences in morphological knowledge: A nonce-word study

**DOI:** 10.3389/fpsyg.2023.1136337

**Published:** 2023-04-26

**Authors:** Ewa Dąbrowska, Esther Pascual, Beatriz Macías-Gómez-Estern, Miquel Llompart

**Affiliations:** ^1^Chair of Language and Cognition, Department of English and American Studies, Friedrich-Alexander-Universität Erlangen-Nürnberg, Erlangen, Germany; ^2^Department of English Language and Linguistics, University of Birmingham, Birmingham, United Kingdom; ^3^Institute of Linguistics, Shanghai International Studies University, Shanghai, China; ^4^Department of Social Anthropology, Basic Psychology and Public Health, Universidad Pablo de Olavide, Seville, Spain; ^5^Department of Translation and Language Sciences, Universitat Pompeu Fabra, Barcelona, Spain

**Keywords:** literacy, individual differences, morphological productivity, Spanish, verbal morphology/derivation, imperfect, preterite

## Abstract

Using a nonce-word inflection task, we examine the morphosyntactic productivity of adult native speakers of Spanish who are either beginning to learn to read and write (semi-literates) or have acquired literacy in late adulthood (late-literates), as well as age-matched controls (high-literates). High-literates consistently provided the appropriate form more often than late-literates, who in turn were better than semi-literate participants. Crucially, group interacted with person, number, and conjugation, such that the between-group differences were larger for the less frequent cells in the paradigm, indicating that literacy-related differences are not merely a consequence of the high-literacy group being more engaged or test-wise. This suggests that the availability of written representations may facilitate the acquisition of certain aspects of grammar. We also observed vast individual differences in productivity with inflectional endings. These results add to the growing body of research which challenges the assumption that all native speakers converge on the same grammar early in development.

## Introduction

A number of studies have demonstrated that, contrary to a widely held belief, native speakers do not converge on more or less the same grammar. In fact, considerable differences have been found in individual speakers’ mastery of a variety of complex structures involving subordination, quantifier scope, as well as some aspects of inflectional morphology (for recent reviews, see Dąbrowska, 2012, 2015; Hulstijn, 2015; Kidd et al., 2018). Some, though not all, of the observed differences are related to educational attainment, and these follow a consistent pattern: while highly educated participants typically perform at or close to ceiling, less educated participants show a large amount of variation, some scoring at ceiling, some at or even below chance, and most falling somewhere in between. The rigorous controls employed in these studies show that these differences are not due to stylistic choices, dialectal differences, or linguistically irrelevant performance factors: instead, they reflect genuine differences in linguistic knowledge.

Why should education matter? One obvious, and theoretically rather uninteresting, reason is that educated speakers are likely to have received some instruction in prescriptive grammar and thus learned to avoid grammatical variants of certain constructions that are socially stigmatized, such as double negatives or ‘dangling’ prepositions in English. However, this explanation does not apply to the studies discussed above, which involved comprehension of structures that are not subject to dialectal variation (i.e., there are no dialects of English in which, for example, *the boy that the girl kissed* means ‘the boy that kissed the girl’).

A second, more relevant reason for education-related differences in grammatical knowledge could be the fact that formal education involves considerable exposure to written texts. Written texts tend to be linguistically more complex than spoken texts, in the sense that they are lexically richer (Cunningham and Stanovich, 1998; Johansson, 2008) and contain higher proportions of various complex structures (Miller and Weinert, 1998; Cameron-Faulkner and Noble, 2013). Thus, compared to low-literacy language users, speakers with significant exposure to print will have experienced more tokens of, e.g., relatively infrequent inflectional forms or sentences with non-canonical word order such as passives, and therefore, to the extent that exposure to more types results in greater productivity (*cf.* Bybee, 1995, 2010; Abbot-Smith and Tomasello, 2006; Dąbrowska and Szczerbiński, 2006), are more likely to become productive with such structures.

A third possible reason is that the availability of written representation eases working memory load, in the sense that readers can re-read difficult passages and writers can edit previously produced text. In other words, written representations can act as a ‘processing crutch’ which enables experienced readers to produce and understand sentences they would not otherwise have been able to process; furthermore, through practice in the written medium, such language users may eventually come to be able to process the same structures in the spoken modality as well (the ‘training wheels’ hypothesis: see Dąbrowska, 2021).

Last but not least, the acquisition of literacy may benefit grammatical development by supporting the development of metalinguistic skills, which have been shown to be related to grammatical comprehension and the ability to detect ungrammatical structures (Dąbrowska, 2018; Llompart and Dąbrowska, in press; Winckel and Dąbrowska, under review). Learning to read requires achieving a certain level of metalinguistic awareness; and the availability of written representations facilitates reflection on form by making it available as a lasting entity rather than fleeting sound. Thus, individuals who achieve higher levels of literacy also tend to have better metalinguistic skills than less literate individuals, which could enhance their grammatical abilities.

It is important to note at this point that the low-educated participants in the studies cited above had at least 10 years of formal schooling. This raises the possibility that the education-related differences discussed above would be even larger if we compared highly educated speakers with individuals who were unable to attend school in childhood and hence either never learned to read and write at all or acquired basic literacy only in adulthood.

Illiterate and semi-literate speakers are a difficult population to study. In industrialized countries, everyone goes to school for at least 8 years; thus, the only true illiterates suffer from severe mental impairments or extreme social deprivation. In parts of Africa, Latin America, and Asia there are many people who do not have access to education. However, these individuals usually speak little-studied languages, which makes it difficult to design appropriate tests; and testing them often raises various logistical problems. Moreover, gaining the participants’ trust is particularly important in this population, and this is something that requires a considerable amount of time and effort.

In this paper, we describe a study testing the linguistic abilities of semi- and late-literate adult speakers of Spanish. Literacy rates in Spain were relatively low until the 1970s, when primary education became compulsory for all children and massive adult literacy programmes were launched. The adult literacy rate in today’s Spain is comparable to that of other European countries (98% in adults, 95% in elderly adults). However, the infrastructure for educating illiterates is still in place in some parts of the country, making it considerably easier to reach this group than elsewhere in the developed world.

While there is some work on speech processing and metalinguistic awareness in semi- and illiterate speakers, very little is known about their morphosyntactic abilities. One recent study (Eme et al., 2010) analyzed oral narratives produced by semi-illiterate French-speaking adults and age-matched controls. The researchers observed significant differences between groups in the number of morphological errors and syntactic complexity (mean length of clauses and proportion of complex structures), with effect sizes (Cohen’s *d*) ranging from 0.67 to 1.1, as well as in the ability to produce coherent narratives.

The semi-literate participants in this study had attended school as children but had attained literacy levels below those expected of third-graders. Thus, it is likely that their difficulties with acquiring literacy might have been due to language impairment. To our knowledge, the only published research on the morphosyntactic abilities in adult illiterates who did not learn to read as children because they were unable to attend school for social reasons is a recent study by Dąbrowska et al. (2022), which tested comprehension of object relatives (e.g., *el niño al que la niña empuja* ‘the boy that the girl is pushing’) using a two-alternative picture selection task. Subject relatives (e.g., *el niño que empuja a la niña* ‘the boy that is pushing the girl’) were used as a control condition. The participants were three groups of older Spanish-speaking women: semi-literates, or individuals who are still learning to read; late-literates, that is to say, individuals who learned to read in late adulthood; and high-literates, who learned to read as children, and obtained at least a secondary school diploma. All three groups were at ceiling on subject relatives, showing that they had understood the task, were cooperative, etc. However, there were dramatic differences in the comprehension of object relatives. High-literates achieved relatively high scores (84% correct). The two low-literacy groups, in contrast, performed very poorly, with semi-literates averaging 51% correct (i.e., at chance), while the late-literates were slightly above chance (66% correct).

In this paper, we investigate semi- and late-literate participants’ productivity with Spanish past tense inflections (the preterite and the imperfect). To our knowledge, there is no previous research on morphological productivity in cognitively unimpaired semi- or illiterate speakers. One reason for this may be the widespread belief that children acquire the basic inflectional patterns of their language very early. There is, indeed, evidence that inflectional endings such as case and plural markers on nouns and verb inflections emerge very early. For example, corpus-based studies show that Spanish-speaking children begin to produce tense and agreement markers on verbs between the ages of 1;6 and 2;0, and begin to use these productively (as evidenced, for example, by overgeneralization errors) from about age 2;0 (Clahsen et al., 2002; Montrul, 2004; Soto-Corominas, 2021). Furthermore, error rates in spontaneous speech are very low.

However, these findings are misleading. One reason for this is that many of the correctly inflected forms produced in spontaneous speech may be rote-learned. This is illustrated by a study by Aguado-Orea and Pine (2015). The authors investigated verb use in the spontaneous speech of two Spanish-speaking children between the ages of 2;0 and 2;6. On average, the children produced 1.8 different inflectional forms per verb: in other words, the vast majority of verbs occurred in only one or two inflectional forms. Furthermore, there were large differences in accuracy on forms belonging to different parts of the paradigm. The children were overwhelmingly correct on third person singular (3 s) forms (over 99% correct) and the first person plural (95–97% correct). These are very frequent forms, accounting for 87% of the verb tokens produced by the children. In contrast, error rates on less frequent forms, such as the second and third person plural, were much higher (from 33 to 46%). The overall error rate was 4%. In other words, the very low error rates on the most frequent forms hide much higher error rates in low frequency cells in the paradigm, suggesting that the children had acquired only parts of inflectional system of the language.

A comprehension study by Pérez-Leroux (2005) also raises doubts about the early acquisition of verb inflections. In the study, children were presented with short sentences without an overt subject, e.g., *duerme en la cama* ‘(he/she/it) is sleeping on the bed’ or *duermen en la cama* ‘(they) are sleeping on the bed’ and pictures depicting either one or two participants engaged in the action (e.g., one or two sleeping cats). Their task was to point to the picture that went with the verbal prompt. The younger children (aged from 3;2 to 4;5) were at chance in both conditions (52% correct in the singular and 45% in the plural); older children (aged 4;8–6;6) were slightly above chance (67%) on plural forms, while still at chance (50%) on singular forms. These results suggest that children take much longer to master the verb system than a cursory look at spontaneous speech would suggest.

This impression is confirmed by two studies which used nonce verbs, i.e., novel verbs made up for the purpose of the study. The first of these (Schnitzer, 1996) examined speakers’ ability to use nonce verbs in a variety of forms (present, preterite, imperfect, future, and conditional). The participants were Spanish-speaking adults and older children (aged 10–11). Mean scores ranged from 70 to 90%, with the children achieving slightly higher scores than the adults. Importantly, the scoring criteria used in the study were relatively lenient, in that a response was coded as correct if the participant used an ending that belonged to the correct conjugation and carried the appropriate person and number ending: in other words, substitutions of a different tense or aspect were not counted as errors. The second study (Brovetto, 2002; a subset of the results was subsequently published in Brovetto and Ullman, 2005) elicited third person imperfect forms of nonce verbs from adult native speakers of Spanish. The participants supplied the target form for 66% of the stimuli.^1^ Another 10% were conjugation errors, i.e., substitutions of an ending appropriate for a different conjugation. The remaining 24% of the responses were either incomplete or contained “other transformations” (p. 280). Given the rather heterogeneous nature of these ‘other’ responses, the results are difficult to interpret; however, it is clear that the participants’ performance was nowhere near ceiling. It is worth noting that Brovetto’s participants supplied the inflected forms of real verbs in the imperfect in 98% of the trials. The large discrepancy between performance on real and nonce verbs would support the conjecture that even adults may not be fully productive with such forms.

Thus, producing a past tense form of a novel verb appears to be challenging even for adult speakers, which makes it promising area for studying individual differences in linguistic knowledge, and the possible role of literacy to explaining such differences.

### Spanish verbal morphology

In this study, we use a nonce-verb inflection task to examine the morphosyntactic productivity of adult native speakers of Spanish with different literacy levels. Spanish verbs are marked for person, number, tense, aspect, and mood. There are three classes of verbs (traditionally referred to as ‘conjugations’), distinguished by thematic vowels. First conjugation verbs are characterized by the thematic vowel *a* and constitute the largest class by a large margin: according to Mendoza et al. (2001), 78% of all verbs belong to this group. The second and third conjugations are characterized by the vowels *e* and *i*, respectively, and each accounts for about 11% of all verbs (Mendoza et al., 2001). As shown in Table 1, the paradigms for these two classes share many endings, and the preterite and imperfect endings are identical.

**Table 1:** The indicative forms of Spanish verbs.

	1st conjugation	2nd conjugation	3rd conjugation
Infinitive	*hablar* ‘speak’	*comer* ‘eat’	*vivir* ‘live’
Present			
1s	hablo	como	vivo
2s	hablas	comes	vives
3s	habla	come	vive
1p	hablamos	comemos	vivimos
2p	habláis	coméis	vivís
3p	hablan	comen	viven
Preterite			
1s	hablé	comí	viví
2s	hablaste	comiste	viviste
3s	habló	comió	vivió
1p	hablamos	comimos	vivimos
2p	hablasteis	comisteis	vivisteis
3p	hablaron	comieron	vivieron
Imperfect			
1s	hablaba	comía	vivía
2s	hablabas	comías	vivías
3s	hablaba	comía	vivía
1p	hablábamos	comíamos	vivíamos
2p	hablabais	comíais	vivíais
3p	hablaban	comían	vivían
Future			
1s	hablaré	comeré	viviré
2s	hablarás	comerás	vivirás
3s	hablará	comerá	vivirá
1p	hablaremos	comeremos	viviremos
2p	hablaréis	comeréis	viviréis
3p	hablarán	comerán	vivirán
Conditional			
1s	hablaría	comería	viviría
2s	hablarías	comerías	vivirías
3s	hablaría	comería	viviría
1p	hablaríamos	comeríamos	viviríamos
2p	hablaríais	comeríais	viviríais
3p	hablarían	comerían	vivirían

For the purposes of the following discussion, two additional features of the Spanish verbal paradigm should be noted. First, the different parts of the paradigm differ in transparency. Past imperfect forms can be segmented into morphemes corresponding to the root, thematic vowel, tense-aspect marker and person-number marker:

*habl- -a -ba -is*

speak ThV IMP 2p

‘you (pl) spoke/used to speak’

Preterite forms are less transparent, in that the ending conflates tense, aspect, person, and number (see Table 1). Note, too, that not all first conjugation forms share the thematic vowel *a.* Secondly, while in imperfect, preterite, and present forms tense–aspect–mood (TAM) markers and agreement markers are added to the verb stem (i.e., root plus thematic vowel) or directly to the root, in the future and the conditional the endings are added to the infinitive form (root + thematic vowel + − *r*).

Both preterite and imperfect forms are used to refer to the past. The preterite is prototypically used to describe events which occurred at a particular point in the past (often with adverbials such as *ayer* ‘yesterday,’ *anoche* ‘last night,’ *el año pasado* ‘last year’). The imperfect, in contrast, is used to describe incomplete and habitual actions (typically with adverbs such as *a menudo* ‘often,’ *siempre* ‘always,’ *cada día* ‘every day’).

### The current study

In this study, we examine adult Spanish speakers’ productivity with verbal inflections by presenting them with nonce verbs and inviting them to use these in a different form. Half of the nonce verbs belonged to the first conjugation and the other half to the second conjugation. We elicited the verbs in three person/number combinations (third person singular, first person plural, and second person plural) and two aspects (preterite and imperfect).

We decided on this design (two conjugations, two aspects, and three person-number combinations) because it allowed us to vary the difficulty of the items. As discussed earlier, higher type frequency results in greater productivity; thus, speakers should be more productive, and hence provide the target form more reliably, with first conjugation verbs than with second conjugation verbs because of their higher type frequency. Furthermore, speakers should be more productive with third person singular (3 s) forms than with the first person plural (1p) and more productive with the first person plural than with the second person plural (2p). The reason for this has to do with differences in the frequency of these forms. Cross-linguistically, third person singular forms are far more frequent than first person plural ones, which in turn are more frequent than second person plural forms (Greenberg, 1966). This is also the case for Spanish: according to figures cited by Bybee and Brewer (1980), 3s verbs in Spanish are an order of magnitude more frequent than 1p forms, which in turn are much more frequent than 2p forms. This means that speakers are likely to have heard most of the verbs they know in the third person singular, a relatively large number of verbs in the first person plural, and a much smaller number of verbs in the second person plural. A similar argument can be made for preterite and imperfect forms, although here the difference in frequency is much smaller. (According to the figures provided by Bybee and Brewer, 1980, the preterite is about three times more frequent than the imperfective; according to Bull et al., 1947, the difference is only about 50%).

We tested three groups of participants who differed in the amount of exposure to written language. For the reasons discussed in the introduction, we expected participants with higher levels of literacy to outperform those with lower literacy levels. Furthermore, these differences should be larger for more difficult structures. This is because there is generally more individual variation on more difficult structures, and hence more scope for potential benefits of literacy to become manifest. Thus, we make the following predictions:

1. More literate participants will achieve higher scores overall.
2. All three groups should do better on first conjugation than on second conjugation verbs.
3. All three groups should achieve the highest scores on 3s forms followed by 1p forms and lowest scores on 2p forms.
4. All three groups should do better on preterite than on imperfect forms.
5. The differences between semi-literates, late-literates and high-literates should be more pronounced on the more difficult forms: in other words, we predict an interaction between literacy and the linguistic factors listed above.

In addition to the nonce-word inflection task, our participants also completed a non-verbal IQ test (Raven’s Coloured Progressive Matrices, CPM). Since schooling improves intelligence (Ritchie and Elliot Tucker-Drob, 2018), including IQ as an additional predictor makes it possible to determine whether any observed differences between the groups are attributable to general cognitive ability rather than linguistic experience *per se*.

## Method

### Participants

In industrial countries, illiteracy carries considerable stigma. Thus, one of the most difficult aspects of conducting research on the effects of literacy is finding participants and persuading them to participate. We were able to overcome this obstacle by taking advantage of the extensive network of contacts that the third author had developed over almost 30 years of research in Polígono Sur, a severely disadvantaged neighborhood in Seville, Southern Spain. This enabled us to build a strong relationship with the director and teachers of the Polígono Sur Adult Continuing Education Centre (CEPer) and obtain their support in recruiting adults with low literacy skills. Thus, the experimental participants were enrolled in adult literacy classes at the center. They were all late middle-aged and elderly women who had not learned to read as children for social reasons: they were either unable to attend school at all or attended only briefly and irregularly. In most cases this was because they had to look after younger siblings and/or other family members while their parents worked; a few had to work from a very young age to help support their families. Most of the participants had attended classes at the Polígono Sur for two or more years; however, attendance was often quite irregular due to various family commitments. Information about the participants’ ages is provided in Table 2.

**Table 2:** Participants’ age by group.

Group	Mean	SD	Median	Interquartile range	Range
Semi-literates (*N* = 20)	68.6	8.4	68	62–74	52–89
Late-literates (*N* = 13)	70.9	8.4	72	68–78	49–79
High-literates (*N* = 14)	67.7	6.0	68	63–72	59–77

Based on teacher assessment and the level of the literacy courses they were attending, we divided the participants into two groups. The semi-literate participants (*N* = 20) knew most or even all letters, could read simple words and write their name, but were unable to read texts with understanding. The late-literate participants (*N* = 13) could read simple texts; some even reported reading whole novels. We decided to rely on the teachers’ assessment of the students’ literacy levels rather than a formal literacy test as we felt that, in this population, this would produce more reliable results. We had originally planned to include only participants aged below 75. However, several older women, including one 89-year-old, insisted on participating, so we decided to include them in the sample and control for age statistically. In addition, we recruited a control group of 14 aged-matched female participants through a university of the third age (‘Aula de Mayores’) in the Seville area. All of these participants learned to read as children and held at least a secondary school diploma; most had a university degree.

### Stimuli

The stimuli consisted of 26 nonce verbs (24 experimental items and 2 practice verbs). Half of these (12 experimental items and 1 practice verb) belonged to the first conjugation (with the thematic vowel *a*) and the other half to the second conjugation (with the vowel *e*). Within each conjugation, half of the nonce verbs occurred in a grammatical context calling for the preterite and the other half in a grammatical context associated with the imperfective (see below). Of the 12 verbs for each aspect, four (two *a* and two *e*) occurred in each person-number combination (third singular, first plural, and second plural).

We had originally planned to use the nonce verbs from Brovetto (2002). However, after consultation with adult literacy teachers, we changed some of the verbs to make them easier to pronounce: for example, *josoblar* and *praltar* were replaced with *pojar* and *gicar*. A list of verbs used in the experiment and their meanings is provided in the Supplementary material.

In addition to the nonce-word task, we also administered a measure of non-verbal IQ (Raven’s Coloured Progressive Matrices, CPM, Raven et al., 1990, 1996). The CPM is designed for testing children, the elderly, and mentally handicapped adults. It comprises 3 sets of 12 visual problems of increasing difficulty. Each problem consists of the picture of a colored rectangular pattern with a missing part, which the respondent needs to identify from an array of 6 options printed beneath it.

### Procedure

Participants were tested individually in a familiar location (the library at the CEPer for semi- and late-literate groups, a nearby cafe or in a few cases a private home in the case of the high-literates). The high-literates were tested in a single session and took an average of 45 minutes to complete the tasks. The semi- and late-literate participants tended to take much longer, around 2 hours, and in a few cases it was necessary to break up the testing into two sessions, with the second session usually taking place 1–3 days later. The break always occurred between tasks rather than in the middle of a task.

Each session began with an informal chat to establish rapport. Once the participant was relaxed, the experimenter explained the tasks and asked if the participant was happy to continue. There were four tasks, always administered in the same order:

1. Background interview, which contained questions about age, literacy level, formal education, reading ability, and reading habits;
2. The relative clause comprehension task (discussed in Dąbrowska et al., 2022);
3. Nonce-verb production;
4. Raven’s Coloured Progressive Matrices (CPM).

There were short breaks between tasks and whenever the participant needed them. The experimenter provided encouraging feedback throughout the entire session and asked if the participant was happy to continue before beginning a new task. Most participants were relaxed, chatty, and enthusiastic about participation: they appeared to be delighted at the attention they were getting. The experimenter took notes of the participants’ responses, including signs of hesitation (long pauses, self-repetitions, etc.), and audio and/or video-recorded the entire session for later checking.

Inflecting nonce words in an experimental setting is obviously not something that our participants do in their daily lives. This raises the problem of how to make the task non-threatening and to explain it to the participants in terms that would make sense to them. We decided to present the task as a game with words that were supposedly used in Lepe — a village in Andalusia whose inhabitants are supposed to be somewhat obtuse and are the subject of innumerable jokes. The experimenter began by asking the participant if she knew any Lepe jokes. Following this, they exchanged one or two, providing a short break before the actual task and ensuring that the participants were comfortable. Since low-educated participants sometimes refuse to inflect unfamiliar words because they do not know what they mean, we initially presented each verb in the infinitive with a simple definition. The experimenter then modeled the verb in a present tense form and a past tense form (either the imperfect or the preterite, depending on the condition), and then began a sentence with a different subject. The participant’s task was to complete the sentence orally, the prompts also being provided only orally. The prompts contained adverbial modifiers strongly associated with either the preterite (*ayer* ‘yesterday,’ denoting a particular point of time in the past) or the imperfect (*antes siempre* ‘in the past always,’ strongly implying a past habitual action). Examples of test items are provided in (1) and (2) below.

(1) Prompt for the imperfective:

*Resulta que en Lepe dicen ‘tarrer’ para decir “cubrir una cosa con una tela.”*

‘It turns out that in Lepe they say *‘tarrer’* to mean “to cover something with a cloth.”’

*Entonces yo soy de Lepe y digo: Nosotros ya no tarremos más. Antes siempre tarríamos y Diego también…*

‘Then, I am from Lepe and I say: We do not tarrer any more. In the past we always tarrered and Diego also…’ (target: *tarría*).

(2) Prompt for the preterite:

*En Lepe dicen ‘lendar’ para decir “fregar usando un estropajo o cepillo.”*

‘In Lepe they say ‘lendar’ to mean “to scrub using a scouring pad or brush.”’

*Entonces yo soy de Lepe y digo: Hoy yo no lendo. Ayer lendé mucho y vosotros también…*

‘Then, I am from Lepe and I say: Today I do not lendar. Yesterday I lendarred a lot and you (pl) also…’ (target: *lendasteis*).

While reading the prompts, the experimenter gestured to highlight the present/past contrast (pointing downwards for the present and over her shoulder for the past) and the person and number of the subject (leaning slightly backwards and pointing toward herself when the subject was the first person singular; gesturing away to an imaginary third person for 3s subjects, toward the participant and then away for 2p, and toward herself and then the participant for 1p).

The task began with two practice verbs, which were not scored. These were followed by the 24 test items presented in semi-random order with the constraint that no two items with the same person, number, and aspect were adjacent to each other. There were two different experimental lists differing in the order; in each group, half of the participants were given list 1 and the other half list 2. If the participant hesitated or produced more than one response, the experimenter repeated the prompt until the participant committed to a specific form. All responses were recorded, but only the final one was scored. In a few cases, participants refused to commit to one form, claiming that two different responses were equally good. In such cases, for the sake of consistency, we scored the final response.

## Results

### Coding scheme

Consider the responses we observed for the third person singular imperfect form of the second conjugation of the nonce verb *tarrer*. As shown in Table 3, we recorded 23 different responses to this verb. This is by no means atypical: the number of distinct responses for individual items ranged from 11 to 33.

**Table 3:** Individual responses for 3s imperfect of *tarrer.*

	Verb	Semi	Late	High	Nonce	Stem	Person	Number	Conj.	Tense	Aspect
1	tarría	2	3	2	1	1	1	1	1	1	1
2	terría		1		1	0	1	1	1	1	1
3	tarraba	1		1	1	1	1	1	0	1	1
4	tarriaba	2	1		1	0	1	1	0	1	1
5	tarríaba	1		1	1	0	1	1	0	1	1
6	tarreaba	2	2		1	0	1	1	0	1	1
7	terriaba		1	1	1	0	1	1	0	1	1
8	tarrió	1	2	2	1	1	1	1	1	1	0
9	carrió		1		1	0	1	1	1	1	0
10	tarró		1	3	1	1	1	1	0	1	0
11	tarré	1			1	1	0	1	0	1	0
12	taré	1			1	0	0	1	0	1	0
13	tarre	1			1	1	1	1	1	0	na
14	tarrea	2		1	1	0	1	1	0	0	na
15	tadea	1			1	0	1	1	0	0	na
16	tarriámo(s)	1			1	0	0	0	0	0	na
17	tarrerá	1		2	1	1	1	1	1	0	na
18	tarreará	1			1	0	1	1	1	0	na
19	restaba	1			0	na	na	na	na	na	na
20	estaría	1			0	na	na	na	na	na	na
21	arrast(r)ó	1			0	na	na	na	na	na	na
22	nastaras		1		1	0	0	1	0	0	na
23	tarrá			1	1	1	1	1	0	0	na

Note: Columns 3–5 provide information about the number of times each form was attested in each group. Columns 6–12 show how these forms were coded.

The first type response, *tarría*, is the expected form and was produced by 6 out of our 47 respondents. The second response type also contains the target ending, but the vowel in the stem has been changed to [e]. The third response type involves overgeneralization of the first conjugation ending -*aba* to a second conjugation verb. The next four response types (4–7) involve overgeneralization of *-aba* as well as insertion of the vowel [i] or [e]. (These errors will be discussed in more detail in the subsection ‘Additional observations’ below.) Response type 8 contains a third person ending for a past tense of a second conjugation verb, but the aspect is incorrect (preterite rather than imperfect). Response type 9 contains the same type of error in addition to a change in the initial consonant of the stem. Response type 10 contains the correct stem and a third person singular preterite ending for -*ar* verbs: it thus involves both an aspect error and a conjugation error. Response type 11 seems to involve the use of the first person singular preterite form: thus, the stem is correctly marked for number and tense, but the person, conjugation and aspect are wrong. Response type 12 contains the same errors in addition to a phonological error in the stem, namely, the substitution of [ɾ] for [r]. The response in 13 contains the correct stem and a third person singular present tense ending: thus, the person and number are correct, but the tense is wrong. Response type 14 contains the correct stem followed by the thematic vowel for second conjugation verbs followed by a first conjugation 3 s present tense ending: thus, it contains the correct person and number marking, but the stem, the conjugation and the tense are incorrect. Response type 15 is identical to 14 except that it contains an additional phonological error (substitution of [ð] for [r]). In response type 16 the participant appears to modify the last nonce form in the prompt by shifting the stress; the resulting form contains the root, the unstressed vowel [i] and the first person plural present ending. In response type 17, the third person singular ending -*a* appears to have been added to the infinitive rather than the root, resulting in a form that would be the correct third person singular future tense form. (These types of responses will be discussed in more detail in subsection ‘Additional observations’ below.) Response type 18 is the same as 17, except that an extra vowel was inserted before the infinitive ending. Response types (19)–(21) involve substitutions of real verbs for the nonce verbs. Response (22) appears to involve the substitution of a different stem (*nastar*) and the addition of the second person singular present indicative ending. Finally, the response in (23) cannot be related to any existing inflection: it appears to be a one-off innovation. Responses of this kind will also be discussed in more detail in subsection ‘Additional observations.’

As these examples illustrate, there are many ways in which a response could deviate from the target, and a single response often contained more than one error. Partly because of this, some responses were difficult to classify, since it was not always clear which form the participant was trying to produce. In order to capture these complexities, we conducted a two-stage analysis of the data. We first coded each response for seven different categories as follows:

- Nonce: 1 if the participant produced a nonce verb, 0 if she substituted a similar-sounding real verb (in the latter case, all remaining categories were coded as NA);
- Stem: 1 if participant produced the correct stem, 0 if she added, deleted, or permuted segments;
- Person: 1 if the form matched the person in the prompt (1st, 2nd, 3rd), 0 otherwise;
- Number: 1 if the form matched the number in the prompt (singular after a singular subject, plural after a plural subject), 0 otherwise;
- Conjugation: 1 if the participant produced an ending from the correct conjugation, 0 otherwise;
- Tense: 1 for a past tense response (including periphrastic forms such as *he hicado*), 0 for a non-past form;
- Aspect: 1 if the participant produced the preterite after a prompt with *ayer* (‘yesterday’) or the imperfect after a prompt with *antes siempre* (‘in the past always’); 0 if vice versa; NA if the participant produced a non-past form.

This coding scheme enabled us to provide a systematic description of different types of errors. The entire dataset was independently coded by two of the authors; any discrepancies that resulted were resolved through discussion. When a response was ambiguous, participants were given the benefit of the doubt. For example, *baltamos*, offered after a 1p imperfect prompt with the verb *baltar* (target: *baltábamos*), could be interpreted as either a first person plural present or preterite form. In this case, we assumed that the participant produced the preterite, and hence the correct tense (past), but incorrect aspect. Likewise, *tarrerá* and *tarreará* (response types 17 and 18 in the table above) were coded as having the correct person and number, since they could be interpreted as 3s future tense forms. Columns 6–12 in Table 3 above illustrate how the coding scheme was applied to the different responses for the verb *tarrer*.

For the purposes of statistical analysis, we then recoded each response as target (1) or non-target (0), adopting a relatively strict criterion: to be classified as target, the response had to have the correct person, number, conjugation, tense, and aspect implied by the prompt. However, we ignored phonological distortions of the stem. The reason for this is that we were interested in grammar, not phonology.^2^ Furthermore, since many of our participants were elderly, we cannot rule out the possibility that some distortions of the stem might be due to hearing impairment. Thus, for the responses given in Table 3, response types 1 and 2 were coded as target, and all the remaining ones as non-target.

### Descriptive statistics

Table 4 shows the percentage of responses coded as correct for each of the seven criteria separately. With the exception of nonce-verb status and tense, where all three groups performed very similarly, the responses show a clear pattern: the high-literates consistently provided an appropriate form more often than the late-literates, who in turn were better than the semi-literate participants. Importantly, however, the semi- and late-literate participants made the same kinds of errors as the controls: i.e., there were no error types which occurred only in the lower-literacy groups.

**Table 4:** Percentages (and SDs) of correct responses by feature/coding criterion.

	Semi-literates	Late-literates	High-literates
Nonce verb	96 (4)	98 (5)	99 (2)
Correct stem	60 (15)	79 (14)	95 (7)
Person	65 (16)	81 (15)	98 (5)
Number	86 (15)	94 (8)	100 (0)
Conjugation	68 (12)	71 (10)	81 (11)
Tense	74 (26)	79 (17)	75 (33)
Aspect	68 (12)	72 (17)	80 (19)

**Figure 1:**
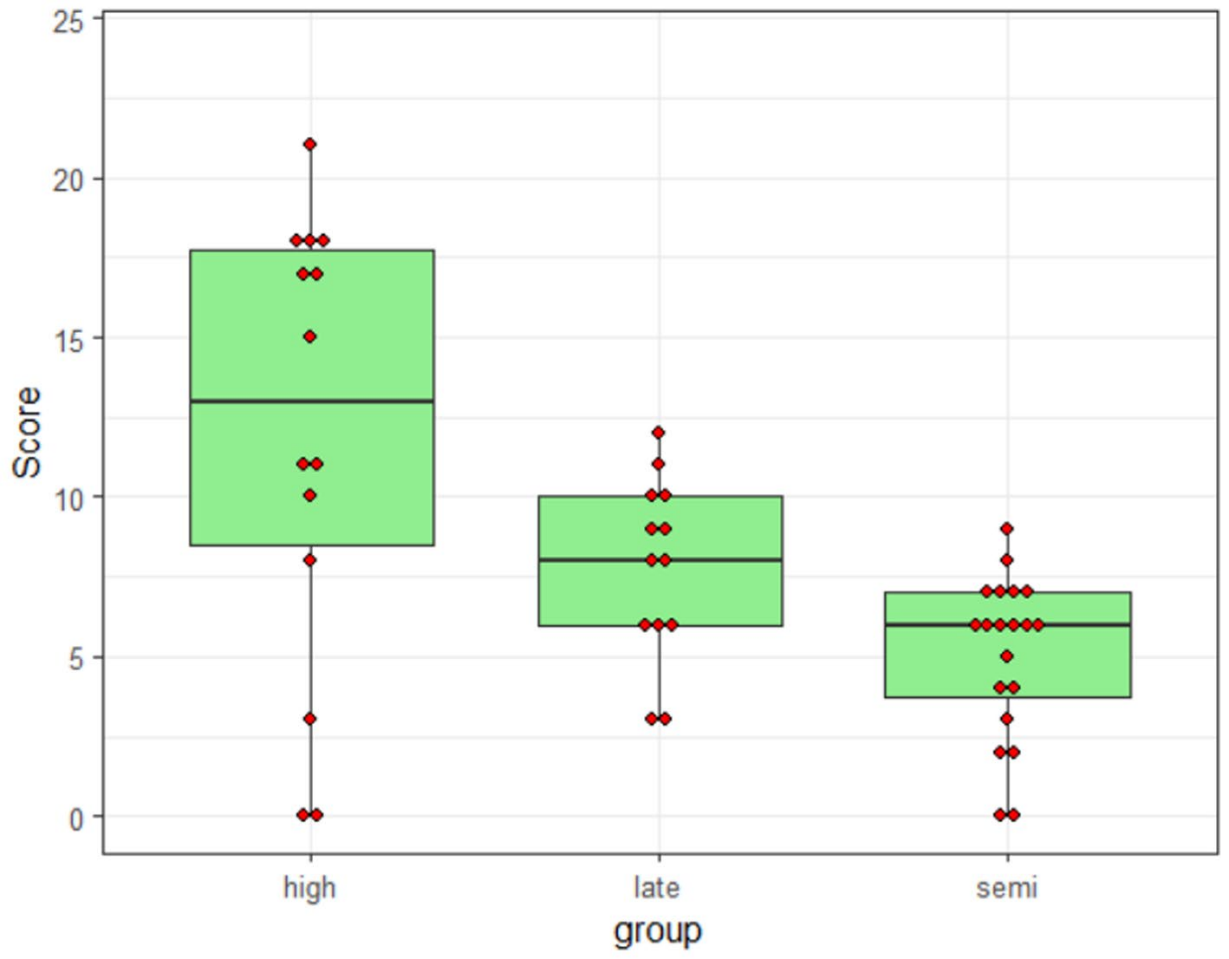
Distribution of individual scores.

Table 5 provides information about percentage of target responses in each group and condition using the scoring procedure described above (i.e., a response was scored as correct if the participant supplied a form that was correctly marked for person, number, conjugation, tense, and aspect). Overall, the control participants supplied the target response in 50% of the trials; for the late- and semi-literate participants, the corresponding figures were 32 and 21%, respectively. It is important to note, however, that the group means mask large individual differences: as shown in Figure 1, individual scores ranged from 0 to 88% correct (i.e., 21/24).

**Table 5:** Percentages (and SDs) of correct responses per group and condition.

	3s -*ar*	3s *-er*	1p -*ar*	1p -*er*	2p -*ar*	2p -*er*
High-literates						
Pret	61 (45)	57 (47)	46 (41)	25 (33)	43 (43)	50 (44)
Imp	64 (41)	25 (33)	64 (46)	50 (44)	57 (47)	54 (50)
Late-literates						
Pret	42 (40)	46 (43)	69 (38)	12 (22)	12 (30)	4 (14)
Imp	58 (34)	31 (38)	50 (50)	23 (39)	30 (38)	12 (30)
Semi-literates						
Pret	38 (43)	25 (34)	65 (43)	10 (26)	0 (0)	0 (0)
Imp	55 (43)	8 (24)	30 (44)	8 (18)	13 (32)	3 (11)

### Regression analysis

For the statistical analysis, only nonce-verb responses were included (i.e., substitutions of real verb forms were treated as missing values). We submitted the trial-by-trial data to a generalized linear mixed-effects model with a logit linking function (lme4 package 1.1–27.1, Bates et al., 2015) in R (Version 4. 1. 1, R Core Team, 2017) that had response (1 = target, 0 = non-target) as a categorical dependent variable. The independent variables to be included were group (semi-literate vs. late-literate vs. high-literate), conjugation (first vs. second), verb form (3s vs. 1p vs. 2p), aspect (preterite vs. imperfect), as well as the non-verbal IQ and age of the participants.

Numeric variables (IQ and age) were centered and scaled, and all categorical variables were contrast-coded so that they were centered on zero and the intercept was thus mapped on the grand mean rather than on a particular combination of factor levels. This has the advantage that any observed effects can be interpreted as main effects, similarly to a traditional Analysis of Variance (ANOVA) test (see Llompart and Reinisch, 2017, 2020 for a similar approach). Group was recoded as two different contrasts of interest to which we will henceforth separately refer as ‘HighVsRest’ and ‘SemiVsLate.’ We contrast-coded ‘HighVsRest’ to capture differences in accuracy between the high-literacy group on the one hand and the semi-literate and late-literate groups on the other. Hence, the responses given by high-literate participants were coded as 0.5 and those given by the semi-literate and late-literate groups were both coded as −0.25. “SemiVsLate” was coded to contrast the differences in accuracy between the semi-literate and late-literate groups. Semi-literate responses were coded as −0.5, late-literate responses as 0.5, and high-literate responses were left at 0. As for conjugation, the first conjugation was coded as 0.5 and the second conjugation was set at −0.5. Similarly to group, verb form was also converted into two separate contrasts: 3sVsRest and 1pVs2p. ‘3sVsRest’ was coded to assess differences between verbs in the 3rd person singular, which were coded as 0.5, and forms in the two plural verb forms, both coded as −0.25. ‘1pVs2p’ contrasted the first and second person plural forms. The former was coded as 0.5, the latter as −0.5, and 3 s forms were left at 0. Finally, aspect was contrast-coded with preterite as 0.5 and imperfect as −0.5.

After these recoding procedures, the fixed-effects structure of the model was set to include HighVsRest, SemiVsLate, conjugation, 3sVsRest, 1pVs2p, aspect, IQ, and age, as well as the interactions between the two group variables (HighVsRest and SemiVsLate) on the one hand and the linguistic variables (conjugation, 3sVsRest, 1pVs2p, and aspect) on the other hand. The random-effects structure included random intercepts for participants and verbs and random slopes for conjugation, aspect, and 1pVs2p over participants. These slopes were included because they improved the model’s fit, as assessed through log-likelihood ratio tests. The “bobyqa” optimizer was used to obtain model convergence. The results of the model are provided in Table 6. The model’s marginal and conditional pseudo-*R*^2^ values as obtained by means of the r.squared GLMM function (MuMIn package 1.43.17, Bartoń, 2009) were 0.37 and 0.63, respectively. The data and R code used in the analysis are provided in the Supplementary material.

**Table 6:** Results of the generalized linear mixed-effects model.

	*b*	Std. Error	*z*	*p*
Intercept	−1.29	0.23	−5.59	<0.001
High vs. Rest	2.04	0.76	2.69	<0.01
Semi vs. Late	1.14	0.51	2.22	<0.05
Conjugation	1.50	0.30	4.97	<0.001
3 s vs. Rest	1.45	0.36	4.04	<0.001
1p vs. 2p	1.60	0.38	4.17	<0.001
Aspect	−0.10	0.32	−0.31	0.75
IQ	0.20	0.24	0.83	0.40
Age	0.48	0.18	2.61	<0.01
High vs. Rest × conjugation	−1.37	0.63	−2.18	<0.05
Semi vs. Late × conjugation	−0.95	0.57	−1.69	0.09
High vs. Rest × s vs. Rest	−2.38	0.67	−3.53	<0.001
Semi vs. Late × 3s vs. Rest	−0.74	0.62	−1.19	0.23
High vs. Rest × 1p vs. 2p	−3.53	0.77	−4.57	<0.001
Semi vs. Late × 1p vs. 2p	−1.36	0.76	−1.79	0.07
High vs. Rest × Aspect	−0.44	0.71	−0.63	0.53
Semi vs. Late × Aspect	−0.63	0.61	−1.04	0.30

The model rendered significant effects of HighVsRest, SemiVsLate, conjugation, 3sVsRest, 1pVs2p, and Age. The effects of aspect and IQ were not significant. This confirms that the control groups was overall more accurate than the late-literate and semi-literate groups, and that the late-literate group performed better than the semi-literate group. Furthermore, participants were more accurate with nonce verbs of the first conjugation than with those of the second conjugation, with 3s forms than with 1p and 2p forms, and among the latter two, they performed better in the 1st person plural than the 2nd person plural condition. Finally, the effect of age indicates that older participants obtained higher scores than younger participants.

**Figure 2:**
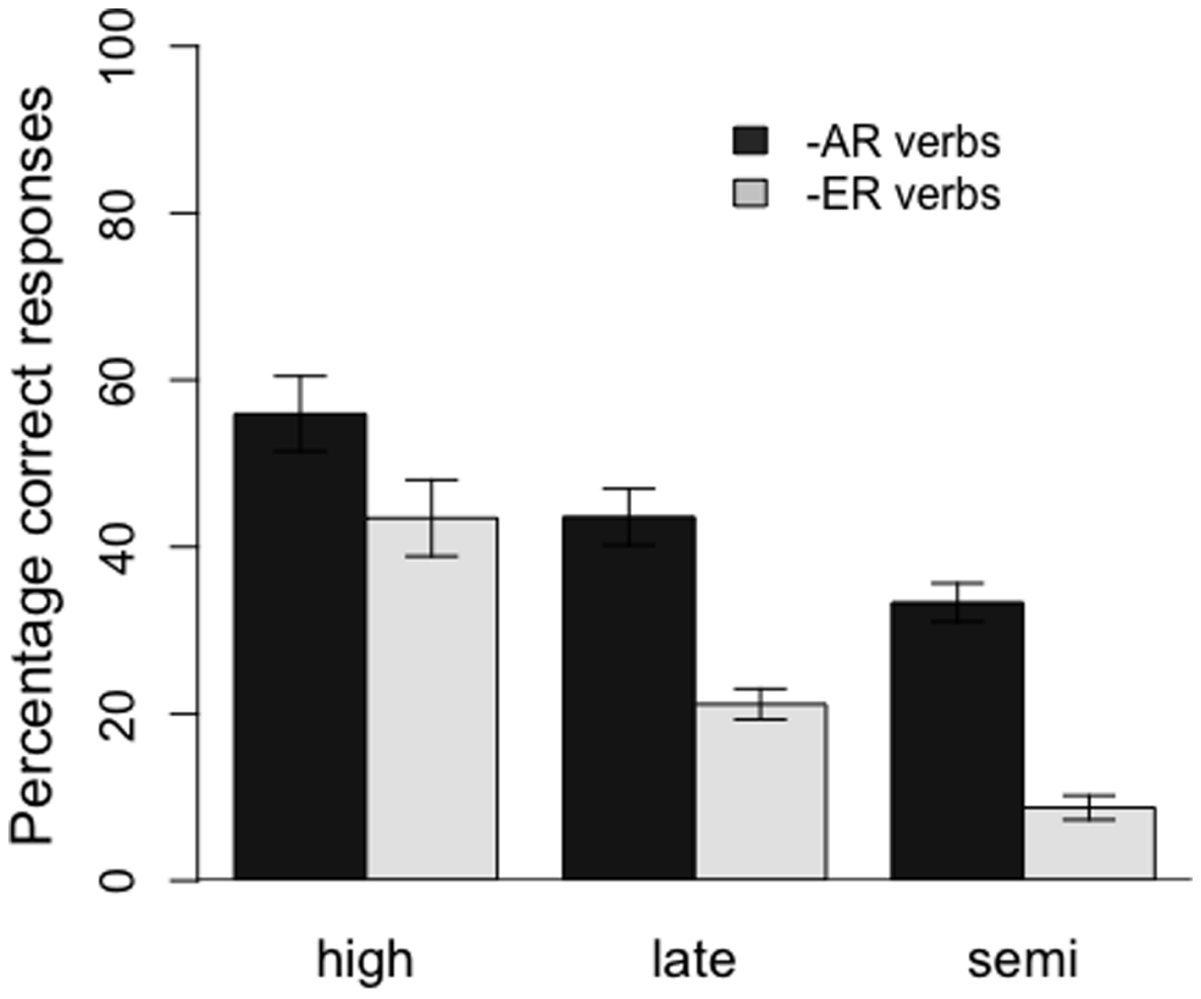
Percentage of correct responses by conjugation (*−ar* verbs and *−er* verbs) for the three groups of participants (high, late, and semi-literates). Error bars represent 1 standard error.

**Figure 3:**
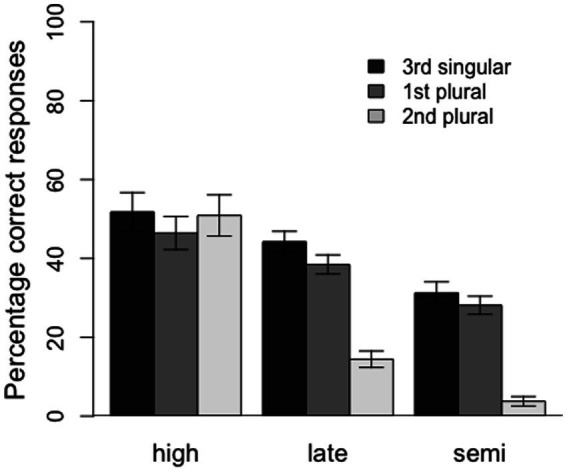
Percentage of correct responses by verb form (3rd singular, 1st plural and 2nd plural) for the three groups of participants (high, late, and semi). Error bars represent 1 standard error.

Furthermore, the model revealed significant interactions between HighVsRest and conjugation, HighVsRest and 3sVsRest, and HighVsRest and 1pVs2p, as well as marginally significant interactions between SemiVsLate and conjugation and SemiVsLate and 1pVs2p. The interactions between the group variables and conjugation suggest that the effect of conjugation was larger for the semi- and late-literate groups than for the control group and that this effect was also larger for semi-literates than for late-literates (see Figure 2). In a similar vein, the interactions between the group variables and 1pVs2p indicate that the difference in accuracy between the 1st and 2nd person plural forms was also larger for the semi- and late-literate groups than for the control group, and larger for the semi-literates than the late-literates. Finally, the interaction between HighVsRest and 3sVsRest indicates a larger difference between 3rd person singular forms and plural forms for the semi- and late-literate groups than for the control, high-literate group. For this comparison, however, there was no difference between the semi- and late-literate groups. The latter three interactions can be seen in Figure 3.

### Follow-up analysis: Group and non-verbal IQ

One interesting finding reported by Dąbrowska et al. (2022) was that non-verbal IQ was a significant predictor of comprehension of object relatives. Moreover, IQ interacted with group, such that IQ had an effect over and above group in the late-literate group only; in other words, learning to read late in adulthood had a larger effect on the comprehension of object relatives in participants with higher IQs. The analysis reported above did not include interactions between IQ and the group variables, as entering them into the model resulted in overfitting and major convergence issues. Therefore, to assess these interactions, we fitted a second model which included only the group variables, IQ, and the interactions between the two group variables and IQ. The model also included random intercepts for participants and verbs and random slopes for SemiVsLate over verbs, as these improved model fit. In contrast to Dąbrowska et al. (2022), the effect of IQ was not significant (*b* = 0.50; Std. Error = 0.32; *z* = 1.55; *p* = 0.12) and neither were the interactions between HighVsRest and IQ (*b* = 0.96; Std. Error = 1.17; *z* = 0.82; *p* = 0.41) or between SemiVsLate and IQ (*b* = 0.12; Std. Error = 0.49; *z* = 0.26; *p* = 0.80). This indicates that there was no effect of IQ on accuracy with the nonce verbs above and beyond the effect of group.

To determine whether the groups differed in IQ, we conducted an additional multiple regression analysis with individual scores in the CPM as dependent variable and HighVsRest and SemiVsLate as predictors. The HighVsRest contrast was significant (*b* = 13.09; Std. Error = 2.00.; *t* = 6.56; *p* < 0.001), indicating that IQ scores for the control high-literacy group (*M* = 30.64, *SD* = 2.31) were indeed higher than those of the other two groups. By contrast, the effect of SemiVsLate was not significant (*b* = 2.05; Std. Error = 1.66; *t* = 1.32; *p* = 0.22), which suggests that the IQ scores for late-literates and semi-literates were not substantially different (late-literates: *M* = 21.85, *SD* = 5.55; semi-literates: *M* = 19.80, *SD* = 5.22).

### Additional observations

The participants were very engaged with the task and appeared to enjoy it, or at least they regarded it as good mental exercise: some even explicitly commented on it being good for them. Furthermore, they had clearly understood the instructions. They were aware that they were supposed to complete the sentence using the novel verb: responses involving substitution of real verbs, which are frequently observed in experiments with young children, were rare (less than 5% of all responses in the semi-literate group and less than 2% in the late-literate group). Furthermore, they clearly understood that they were supposed to use the verb in a different form and that the form depended on the linguistic context in which the verb appeared. However, they were often unsure what the correct response should be. We know this because they sometimes explicitly indicated that they lacked certainty, paused for 4 seconds or more before giving an answer, or changed their mind. For example, in response to the prompt in (2), one participant first volunteered *lluceaba*, then self-corrected to *lluzaba*, and finally reverted to *lluceaba*. Another participant first responded *lechamos*, then *lesté*, and eventually settled on *llustó*. A third participant first produced the target response (*llució*) and immediately followed this with the form used in the prompt (*llucimos*; it is not clear whether this was a self-correction or whether she was simply reminding herself what the verb was), and finally decided on the 3 s present (*lluce*). Such hesitations occurred in 9% of the responses produced by the high-literates; for the late- and semi-literate groups, the corresponding figures were 17 and 23%, respectively.

As evident from the preceding discussion, our participants made a variety of errors. Most of these involved use of a form which was incorrectly marked for person, number, tense or aspect, an ending associated with another conjugation, or some combination of these errors. Some non-target forms, however, involved other kinds of transformations which offer insights into the nature of our participants’ difficulties with inflecting novel verbs.

Participants often produced forms consisting of the infinitive followed by what appear to be the present tense or, less frequently, imperfect endings: for example, *corberá* or *corbería* were produced in response to a prompt calling for the 3 s preterite of *corber* (see also responses 17 and 18 in Table 3 above), and *gerceremos* or *gerceríamos* as the 1p preterite of *gercer*. In most cases, this resulted in forms that correspond to what would have been the future (*corberá, gerceremos*) or the conditional (*corbería, gerceríamos*) forms of the relevant verbs. There were a total of 129 such errors, accounting for 11% of all responses. They were produced by 15 out of 47 participants (4 from the semi-, 6 from the late-, 5 from the high-literate group).

These responses could be interpreted in two ways: either the participants intended to produce future/conditional forms, or they intended to produce a present or past tense form, but failed to correctly identify the stem of the nonce verb: in other words, these responses could involve segmentation errors. The majority of the forms consisting of an infinitive and a person affix are well-formed future and conditional forms, which would support the first interpretation. Furthermore, one participant also produced a periphrastic future (*vais a jasar*, ‘you are going to jasar’), and another participant commented that she wasn’t sure if the target form was supposed to refer to a past or a future action. Thus, it appears that at least some of these forms were intended as future tense forms. On the other hand, it should be noted that time reference was clearly marked in the prompt: the sentence began with an adverbial referring to the past (*ayer* ‘yesterday’ or *antes* ‘before’/‘in the past’); moreover, the target verb was coordinated with another verb in the past tense form immediately preceded by the adverb *también* (‘also’), both of which create strong expectations that the following form should be past: a conditional form in this context is positively odd -- and conditionals made up of 12% of these responses. Furthermore, a substantial minority (almost 30%) of these infinitive stem responses contained the suffixes *-amos* (e.g., *naleramos*), *-éis* (e.g., *vantaréis*), or *-ó* (e.g., *gicaró*), which occur in the present tense or past tense paradigm but not in the conditional or future: in other words, they are not well-formed future or conditional forms, and thus most likely involve segmentation errors.

Two other types of responses strongly suggest that our participants often did not know how to apply the correct inflectional pattern. The first of these are stem augmentation errors which involve the insertion of an additional vowel (in most cases [e], but occasionally [i]) at the end of the stem before the ending. Such insertions occurred with verbs belonging to both conjugations, although most likely for different reasons.

Spanish has two moderately productive suffixes, *-ea(r)* and its colloquial variant -*ia(r)*, which are used to derive verbs from nouns and adjectives. The suffix *-ea(r)* also occurs in many borrowings (e.g., *faxear*, ‘to fax,’ *emailear*, ‘to email’; *chequear*, ‘to check’). Thus, when speakers insert [e] (or, less frequently, [i]) before the thematic vowel in a first conjugation verb, they are subsuming the new root into an existing pattern, and perhaps marking it as non-canonical.

With second conjugation verbs, the reasons for the insertion are quite different. As explained earlier, [e] is the thematic vowel for the second conjugation; the vowel [i] is also found in many forms (see Table 1). However, our participants sometimes inserted these vowels where they did not belong, producing forms such as *vanteamos* as the 1p imperfect of *vanter* (target: *vantíamos*) and *lendeábamos* as the 1p imperfect of *lender* (target: *lendíamos*). Both of these examples involve a combination of a first conjugation ending with the second conjugation thematic vowel: in other words, they are blends containing features of both conjugations. Such forms are innovative in the sense that they are not licensed by the rules of the language (the thematic vowels *e* and *i* cannot co-occur with the first conjugation endings -*amos* or -*ábamos*), although they are arguably motivated by existing rules.

As shown in Table 7, such insertions were much more frequent in the late- and semi-literate groups than in the high-literates. Furthermore, in all three groups, they occurred predominantly with second conjugation verbs. This is striking, given the fact that the combination of a second conjugation thematic vowel with a first conjugation ending is ungrammatical, whereas vowel insertions with first conjugation verbs can be thought of as generalizations of an existing pattern.

**Table 7:** Number and percentage of stem augmentation responses.

	1st conj.		2nd conj.	
	*N*	%	*N*	%
High	6	3.6	13	7.7
Late	13	8.3	29	18.6
Semi	20	8.3	49	20.4

The stem augmentation errors discussed above were relatively systematic, in the sense that many participants produced such forms, typically on several occasions. Our participants also produced other nonstandard forms which are better described as one-off innovations. Some of these could also be blends of two different forms, e.g., *lebadó* (supplied as the third person singular form of *lebar*) was perhaps produced by applying two different patterns simultaneously: adding the ending for the past participle, which would have resulted in *lebado* (with stress on the second syllable) and at the same time adding the normal ending for the third person preterite (a stressed *-ó*).

Other innovations (*cf.* Table 8) are more difficult to explain. However, no matter how they were derived, their existence shows that participants were clearly struggling to produce the correct inflection. Although such one-off innovations were relatively rare, they are considerably more frequent in the late-literates and especially the semi-literates (1.3 and 2.6%) than in the high-literate group (0.2%), again suggesting the former two groups were struggling more than the high-literate participants.

**Table 8:** One-off innovations recorded in the experiment.

Condition	Verb	Target	Innovative form	Group
3s imp	*tarrer*	*tarría*	*tarrá*	High
3s imp	*gicar*	*gicaba*	*gicabar*	Late
3s imp	*tarrer*	*tarría*	*tarrío*	Semi
3s imp	*gicar*	*gicaba*	*gicababa*	Semi
3s pret	*nalar*	*naló*	*nalaló*	Late
3s pret	*nalar*	*naló*	*nalón*	Semi
3s pret	*lebar*	*lebó*	*lebadó*	Semi
1p imp	*sardar*	*sardábamos*	*saliáma*	Semi
2p imp	*nestar*	*nestabais*	*nestabar*	Late
2p imp	*maver*	*mavíais*	*mavevía*	Semi
2p pret	*plerer*	*pleristeis*	*plerero*	Late
2p pret	*plerer*	*pleristeis*	*plerera*	Late
2p pret	*lendar*	*lendasteis*	*lendrea*	Late
2p pret	*plerer*	*pleristeis*	*pleriblo*	Semi
2p pret	*vantar*	*vantasteis*	*vantá*	Semi

## Discussion

### Literacy effects

As predicted, we observed large differences between groups, with the high-literates supplying the target endings more reliably than the late-literates, who in turn were more accurate than the semi-literate participants. Furthermore, also in accordance with our predictions, all three groups performed better on first conjugation than second conjugation verbs, and better on third person singular inflections than on the first person plural, which in turn was easier than second person plural. Crucially, the effects of conjugation and person/number condition were more pronounced in the two low-literacy groups, and in particular in the semi-literates, than in the control participants. Given the very large individual differences in performance within groups and the relatively small sample size, it is all the more surprising that we observed significant effects of literacy.

Contrary to our predictions, we observed no significant effect of aspect and no interaction between aspect and group. This could be because the difference between the frequency of preterite and imperfect forms is relatively small. Alternatively, imperfect forms might benefit from the fact that they are morphologically more transparent than the preterite, which could facilitate drawing parallels between different cells in the paradigm.

In addition to group differences in how often participants supplied the target ending, our more qualitative analysis of participants’ performance provides further evidence suggesting that the low-literate speakers experienced considerable difficulty in producing the correct form of novel verbs. As discussed above, participants belonging to the low-literacy groups often hesitated before providing a response. Sometimes they seemed to ‘grope’ for the right form or overtly expressed lack of confidence; on other occasions they simply took a long time (4 seconds or more) to respond. More strikingly, they produced a relatively large number of innovative forms, which appear to be blends of patterns found in different parts of the paradigm (e.g., insertions of second conjugation thematic vowels before a first conjugation ending) or segmentation errors (e.g., adding a present tense ending to the infinitive rather than the stem). As shown in the preceding section, all of these non-target responses were considerably more frequent in the late- and especially semi-literate participants than in the high-literates.

It is worth noting at this point that, while the nonconventional forms discussed in the preceding section may sound strange, such on-the-fly innovations do occasionally occur in spontaneous speech and have even been induced in an experimental study conducted by Dąbrowska (2017). In this study, native speakers of Polish were simply asked to read aloud a sentence containing the Polish equivalent of the phrase *with 21 policemen* (with the number written in Arabic numerals). Compound numerals ending in one (21, 31, etc.) raise an interesting problem for Polish speakers when they occur in grammatical contexts requiring an oblique case, as in the phrase used in the experiment. This is because both parts of the numeral need to be inflected for case and number. Since the Polish preposition *z* ‘with’ governs the instrumental case, both parts of the numeral (*dwadzieścia*, ‘twenty,’ and *jeden*, ‘one’) have to be instrumental plural. This is unproblematic for the former; however, the numeral *jeden* does not have an instrumental plural — or any plural form, for that matter, for obvious reasons.

The reactions observed in Dąbrowska’s (2017) study were not unlike those of our participants: they hesitated, expressed uncertainty, ‘sounded out’ various options and rejected them, and so on. When pressed, some participants used the regular plural ending; others used the singular ending (which resulted in a number mismatch within the noun phrase), left the numeral *jeden* uninflected, substituted a homophonous indefinite pronoun meaning ‘some’ (which does have a plural); and some produced idiosyncratic forms. Between them, the 21 speakers who participated in the experiment produced 10 different responses.

Clearly, the parallel with Dąbrowska’s study holds only to a certain degree. In the Polish case, there is no conventionalized way of saying things like *with twenty-one policemen* or *behind thirty-one women*. In Spanish, there is a productive paradigm which speakers can fall back on. However, to the extent that the paradigm is not fully mastered by all speakers, the end effect is similar.

In summary, our results provide strong support for the hypothesis that literacy supports the acquisition of morphological patterns. As noted in the introduction, literacy-related differences in grammatical knowledge could be due to several different factors, including explicit teaching and learning of prescriptive grammar, exposure to a wider variety of word types with each morphological ending (which would promote generalization), better metalinguistic skills, or the fact that the availability of written representations may support linguistic development by easing memory load and facilitating comparisons between forms.

It is unlikely that explicit teaching of past tense inflections has a significant influence on the development of past tense inflections. Spanish speaking children learn to produce the past-tense forms of familiar regular verbs in early childhood (Clahsen et al., 2002; Domínguez et al., 2018), and hence these are not taught at school.^3^ This leaves exposure to more verb types, metalinguistic awareness, and facilitating effects of permanent representations on learning as possible explanations for literacy effects in our data. We cannot distinguish between these possibilities on the basis of the results of the current experiment. We note, however, that these explanations are not mutually exclusive, and that it is likely that all three play a role.

### Intelligence

Our results revealed no effect of non-verbal intelligence (assessed using Raven’s Coloured Progressive Matrices) and no interactions between the group variables and CPM. It is worth noting in this connection that the correlation between CPM and performance on the inflection task is relatively strong and highly significant (*r* = 0.63, *p* < 0.001). However, CPM is also correlated with group in our sample, in that highly literate participants have higher IQs: this is unavoidable, since education results in increases in IQ (see Dąbrowska et al., 2022 for further discussion of this issue). Thus, in the regression analysis, the group variable HighVsRest soaks up the variance associated with non-verbal intelligence: in other words, IQ has no effect over and above literacy.

This stands in contrast to the results on the comprehension of object relatives reported in Dąbrowska et al. (2022). For the latter task, there was a main effect of CPM as well as of the two group contrasts; in addition, CPM scores interacted with group such that the late literate participants appeared to benefit from literacy only if they had relatively high IQs. The difference between the two tasks may be due to the fact that inflecting a nonce word involves attending to inflectional endings on individual words (i.e., local cues) and relatively simple grammatical contrasts (first vs. second vs. third person; singular vs. plural; past vs. non-past; habitual vs. specific point in the past). The relative clause task, in contrast, required participants to process complex linguistic stimuli (the head noun plus preposition plus complementizer plus subordinate clause) and map these onto semantic representations of events involving two participants with different roles (agent vs. patient).

### Age

As explained above, age was included in the model as a covariate: since many of our participants were elderly, we wanted to control for possible detriments in performance due to aging. The effect of age turned out to be significant, but the direction of the effect was the opposite from what we expected: older participants were *more* likely to supply the target form than younger participants. Taken at face value, this result suggests that speakers’ linguistic systems continue to improve throughout life — at least if they are exposed to richer input (i.e., written language), which they had been deprived of earlier in their lives. However, the effect could also be attributable to the self-selecting nature of adult continuing education: individuals who are less cognitively fit are less likely to sign up for classes, and hence the older participants are increasingly more unrepresentative of the population from which they come (see Dąbrowska et al., 2022 for further discussion of this issue). Be that as it may, the positive effect of age means that it is extremely unlikely that the low performance observed in our participants was due to dementia.

### Wider implications: Individual differences and the nature of morphological productivity

As we have seen, there were vast individual differences in performance on the nonce-word inflection tasks in all groups, with individual scores ranging from 0 to 21 out of 24 (*cf.* Figure 1 above). This was in spite of the fact that all participants had apparently understood the task: even the semi-literate participants attempted to inflect the nonce verb on 96% of all trials. This finding adds to the growing body of research suggesting that there are considerable individual differences in adult native speakers’ mastery of the grammar of their language.

It is also worth noting that, while the control participants performed much better on the nonce-verb inflection task than the low-literate groups, they supplied the target form in only 50% of the trials. Given the complexity of the system, the number of possible incorrect responses is quite large (*cf.* ‘Coding scheme’ subsection), so getting everything right 50% of the time is still quite a feat. Nevertheless, native speakers are supposed to have full command of at least the basic inflectional patterns of the language — so the reader may be forgiven for being a little skeptical. It is important to note in this connection, therefore, that our results for the high-literate group are comparable to those obtained in earlier studies which investigated Spanish speakers’ knowledge of the verbal paradigm using nonce words. For example, Schnitzer (1996) found that older children and adults supplied the correct form between 70 and 90% of the time. This is higher than the proportion of correct responses observed in our study, but, as explained in the introduction, Schnitzer did not count uses of a non-target tense or aspect as errors. If we apply our scoring method to the results of his first study (conducted in Puerto Rico), the number of correct responses drops to 52%. (It is not possible to adjust the scores for the remaining 4 studies described in the paper, since Schnitzer does not report the number of tense-aspect errors). Brovetto’s (2002) participants provided target forms 66% of the time (see footnote 1 for an explanation of how this figure was arrived at). This is somewhat higher than our control group, but her participants were very highly educated adults, with 19 years of formal education on average (see Brovetto and Ullman, 2005, p. 98).

But if many native speakers do not fully master the rules for forming the preterite and imperfect forms of the verb, how is it possible that they are able to talk about past events in their daily lives, apparently without making errors? Part of the answer is that we do not use nonce verbs in our daily lives: we use verbs that we have heard many times before. For many of these verbs, speakers will be able to retrieve inflected forms from memory. There is independent evidence that speakers store a large number of inflected forms even when these can, in principle, be produced by applying a rule. First, in experimental settings, speakers are much more accurate with real verbs than with nonce verbs. For example, in Brovetto’s (2002) study, participants supplied the target form with real verbs on 98% of the trials. Furthermore, reaction times for nonce verbs were much longer: more than 5 standard deviations longer than those for real verbs.

When a ready-made inflected form is not available, speakers still have several options. They could substitute a verb form with a similar meaning: for example, if they cannot access the correct 1p preterite form, they could substitute it by the 1p imperfect (as our participants often did during the experiment) or the perfect (a periphrastic form consisting of the appropriate form of the auxiliary *haber* and the past participle). This option, of course, is only available if the speaker is able to form the imperfect or the past participle, and the meaning will be slightly different — but it will work in some cases. Another option would be to use a synonymous verb, perhaps one with higher frequency than the verb that best fits the speaker’s communicative intentions — again assuming that an appropriate form of a synonymous verb is available. A third option would be to paraphrase: for example, instead of saying *we forgot,* the speaker could say *I forgot and so did you.* This is not as far-fetched as it might at first seem: for example, Dąbrowska (2017) provides clear evidence that Polish speakers avoid prepositional phrases with the numerals 21, 31, etc. Finally, of course, speakers may use their knowledge of the verbal paradigm and attempt to generate the form they need. When this happens, they will produce the correct form on some occasions. On other occasions, they may end up producing forms which are not licensed by the grammatical system of the language: but if the interlocutors are also unable to generate the correct form, they will not even notice that the form was ungrammatical. And of course, if the interlocutor does know what the correct form is and sees the speaker struggling, he or she may end up producing it for the speaker: in ordinary conversation, interlocutors routinely complete each other’s utterances.

## Conclusion

Human languages in their natural state are spoken or signed. Writing is something that developed relatively recently; it is not universal; and it is clearly a cultural invention rather than something that arose naturally. Because of this, most linguists regard written language as an artificial add-on — or, as Dixon (2016, p. 9) puts it, “an optional extra.” Our results suggest that writing is far more than this. Since both preliterate children and illiterate adults are able to inflect novel words, literacy clearly cannot be regarded as a prerequisite for the acquisition of morphological rules. However, it evidently does help to consolidate the system.

As discussed earlier, literacy effects on morphological factors could be due to the fact that written language is lexically richer, and hence literate speakers are likely to have experienced morphological patterns in a larger number of word types than speakers who cannot read. An alternative explanation is that the acquisition of literacy leads to better metalinguistic skills, which in turn help learners to segment forms and/or analogize across exemplars. Finally, the availability of written representations may support linguistic development in that it eases memory load and facilitates comparisons between forms. Further research will be necessary to distinguish between these explanations. Given the large differences in performance observed even in the control group, it should be possible to test these hypotheses even with literate speakers. For example, in order to test the first two hypotheses, one could examine possible relationships between individual differences in performance on tasks measuring print exposure and metalinguistic abilities on the one hand and the ability to supply the correct forms of novel verbs on the other. To test the ‘training wheels’ hypothesis, one could compare speakers’ performance on a nonce word inflection task administered in the spoken vs. written modality.

## Data availability statement

The original contributions presented in the study are included in the Supplementary material.

## Ethics statement

This study involving human participants was reviewed and approved by the Ethical Review Board of the University of Birmingham (Reference no. ERN_19-1383). Written informed consent for participation was not required for this study in accordance with the national legislation and the institutional requirements. Informed consent to participate in this study was provided by the participants in a recorded oral statement.

## Author contributions

ED: conceptualization, methodology, formal analysis, writing – original draft, project administration, visualization, supervision, and funding acquisition. EP: methodology, investigation, data curation, writing – review and editing, and funding acquisition. BMG-E: investigation, writing – review and editing, and supervision. ML: investigation, data curation, formal analysis, visualization, and writing – review and editing. All authors contributed to the article and approved the submitted version.

## Funding

This project was funded by the Alexander von Humboldt Foundation (grant number ID-1195918) and the ‘Hundred Talents’ programme (grant number 411836).

## Conflict of interest

The authors declare that the research was conducted in the absence of any commercial or financial relationships that could be construed as a potential conflict of interest.

## Publisher’s note

All claims expressed in this article are solely those of the authors and do not necessarily represent those of their affiliated organizations, or those of the publisher, the editors and the reviewers. Any product that may be evaluated in this article, or claim that may be made by its manufacturer, is not guaranteed or endorsed by the publisher.

## Footnotes

^1^Brovetto (2002) analyzed only responses that were either correct or involved conjugation errors: all remaining responses were excluded from the analysis. The overall proportion of target responses given here was computed on the basis of the figures provided in Table 3.64 and footnote 62 on page 280.

^2^It is worth noting that distortions of the stem were much more frequent in the late- and especially semi-literate group than in the high-literates (see Table 3). This echoes earlier findings that illiterates have difficulties with repeating nonce words (see, e.g., Reis and Castro-Caldas, 1997; Petersson et al., 2000), which is usually attributed to less finely-grained phonological representations.

^3^It should be noted, however, that the local dialect uses the pronoun *ustedes* instead of *vosotros* for the second person plural, typically with a second person plural verb (*ustedes vais*), although the third person plural agreement, as in standard Spanish (*ustedes van*), is also attested (see Lara Bermejo, 2012; in our data, third person plural forms accounted for just 2.5% of all responses in the 2p condition). However, our participants would have heard the pronoun *vosotros* in the media and when interacting with speakers of standard Spanish. Furthermore, as explained in the Method section, the experimenter produced the prompt with the appropriate gesture, and explained that *vosotros* means the same as *ustedes* if the participant appeared confused. Furthermore, in all three groups, the majority of participants (14/20 semi-literates, 11/13 late-literates and 14/14 high-literates) produced at least one 2p form after a 2p prompt; in most cases, these forms contained tense and/or aspect and/or conjugation errors. Thus, while dialectal differences might have contributed to the low-literacy participants’ difficulties with 2p inflections, they are unlikely to have affected the results in a substantial way.
