# Supplementary material for: Literacy-related differences in morphological knowledge: A nonce-word study
Source: Front Psychol. 2023 Apr 26;14:1136337. doi: 10.3389/fpsyg.2023.1136337 (PMC10171427; doi:10.3389/fpsyg.2023.1136337)
Supplement: Supplementary file 2 [file Data_Sheet_1.PDF]

Appendix A

Nonce verbs used in the experiment and their meanings

Conjugation	Aspect	Rhyme/ nonrhyme	Person/ number	Nonce verb	Prompt	Definition of nonce verb
-*ar*	IMP	rhyme	3s	*gicar*	*Nosotros ya no gicamos más. Antes siempre gicábamos y Ana también ....* ‘We don't gicar any more. In the past we always gicarred and Ana also ....’	*dejar cosas desordenadas o tiradas en algún lugar* ‘to leave things untidy or lying around somewhere’
-*ar*	IMP	rhyme	1p	*baltar*	*Mateo ya no balta más. Antes él siempre baltaba y nosotros también ....* ‘Mateo doesn't baltar any more. In the past he always baltared and we also ....’	*correr a gran velocidad* ‘to run at high speed’
-*ar*	IMP	rhyme	2p	*nestar*	*Yo ya no nesto más. Antes siempre nestaba y vosotros también ....* ‘I don't nestar any more. In the past I always nestarred and you (pl) also ....’	*hacer caer a tierra; derribar* ‘to bring to the ground; to bring down’
-*ar*	IMP	nonrhyme	3s	*pojar*	*Nosotros ya no pojamos más. Antes siempre pojábamos y Juan también ....* ‘We don't pojar any more. In the past we always pojarred and Juan also ....’	*huir; marcharse con rapidez de algún lugar* ‘to flee; to leave quickly from somewhere’
-*ar*	IMP	nonrhyme	1p	*sardar*	*María ya no sarda más. Antes ella siempre sardaba y nosotros también ....* ‘María doesn't sardar any more. In the past she always sardarred and we also ....’	*llorar; sollozar* ‘to cry; to sob’
-*ar*	IMP	nonrhyme	2p	*gesar*	*Yo ya no geso más. Antes siempre gesaba y vosotros también ....* ‘I don't *ges*ar any more. In the past I always *gesa*rred and you (pl) also ....’	*hacer un espectáculo* ‘make a show’

-er	IMP	rhyme	3s	*tarrer*	*Nosotros ya no tarremos más. Antes siempre tarríamos y Diego también ....*<br>'We don't tarrer any more. In the past we always tarrered and Diego also ....'	*cubrir una cosa con una tela; envolver*<br>'to cover something with a cloth; to wrap'
-er	IMP	rhyme	1p	*lender*	*Isabel ya no lende más. Antes ella siempre lendía y nosotros también ....*<br>'María doesn't lender any more. In the past she always lenderred and we also ….'	*hablar en voz baja*<br>'to speak in a low voice'
-er	IMP	rhyme	2p	*somer*	*Yo ya no somo más. Antes siempre semía y vosotros también ....*<br>'I don't somer any more. In the past I always somerred and you (pl) also ....'	*marchar; andar acompasada y rítmicamente*<br>'to march; to walk steadily and rhythmically'
-er	IMP	nonrhyme	3s	*yoner*	*Nosotros ya no yonemos más. Antes siempre yoníamos y Lucía también ....*<br>'We don't yoner any more. In the past we always yonerred and Lucía also ....'	*gritar; expresar en público, de manera ruidosa, su desagrado hacia algo*<br>'to shout; to express in public, loudly, one's displeasure towards something'
-er	IMP	nonrhyme	1p	*vanter*	*María ya no vante más. Antes ella siempre vantía y nosotros también ....*<br>'María doesn't vanter any more. In the past she always vanterred and we also ….'	*descifrar un enigma o misterio*<br>'to decipher an enigma or mystery'
-er	IMP	nonrhyme	2p	*maver*	*Yo ya no mavo más. Antes siempre mavía y vosotros también ....*<br>'I don't maver any more. In the past I always maverred and you (pl) also ....'	*descansar demasiado*<br>'to rest too much'
-ar	PRET	rhyme	3s	*nalar*	Hoy nosotros no nalamos. Ayer nalamos mucho y Diego también ….<br>'Today we don't nalar. Yesterday we nalarred a lot and Diego also ....'	*coger alguna cosa que huye*<br>'to catch something that runs away'
-ar	PRET	rhyme	1p	*jasar*	*Hoy Sofía no jasa. Ayer ella jasó mucho y nosotros también ....*	*tirar con violencia una cosa*<br>'to throw something violently'

					'Today Sofía doesn't jasar. Yesterday she jasarred a lot and we also ....'	
-ar	PRET	rhyme	2p	*vantar*	*Hoy yo no vanto. Ayer vanté mucho y vosotros también ….*<br>'Today I don't vantar. Yesterday I vantarred a lot and you (pl) also ….'	*hacer un ruido continuo y grave*<br>'to make a continuous and grave noise'
-ar	PRET	nonrhyme	3s	*lebar*	*Hoy nosotros no lebamos. Ayer lebamos mucho y Antonia también ….*<br>'Today we don't lebar. Yesterday we lebarred a lot and Antonia also ....'	*conversar sin previo objetivo ni utilidad, solo por entretenerse*<br>'to talk without prior purpose or usefulness, just to pass the time'
-ar	PRET	nonrhyme	1p	*londar*	*Hoy Felipe no londa. Ayer él londó mucho y nosotros también ….*<br>'Today Felipe doesn't londar. Yesterday he londarred a lot and we also ….'	*dar vueltas*<br>'to turn around'
-ar	PRET	nonrhyme	2p	*lendar*	Hoy yo no lendo. Ayer lendé mucho y vosotros también ….<br>'Today I don't lendar. Yesterday I lendarred a lot and you (pl) also ....'	*fregar; limpiar una cosa con el uso de estropajo o cepillo*<br>'to scrub; t oclean something with a scouring pad or brush'
-er	PRET	rhyme	3s	*corber*	*Hoy nosotros no corbemos. Ayer corbimos mucho y Tomás también ….*<br>'Today we don't corber. Yesterday we corberred a lot and Tomás also ....'	*dar brillo a una cosa; pulir*<br>'to give shine to something; to polish'
-er	PRET	rhyme	1p	*gecer*	*Hoy Sofía no gece. Ayer ella geció mucho y nosotros también ….*<br>'Today Sofía doesn't gecer. Yesterday she gecerred a lot and we also ....'	*observar los movimientos de una persona*<br>'to observe somebody's movements'
-er	PRET	rhyme	2p	*plerer*	*Hoy yo no plero. Ayer plerí mucho y vosotros también ….*<br>'Today I doesn't plerer. Yesterday I plerred a lot and you (pl) also ....'	*tropezar; chocar con algo*<br>'to stumble; to bump into something'
-er	PRET	nonrhyme	3s	*llucer*	*Hoy nosotros no llucemos. Ayer llucimos mucho y Carlos también ….*	*cometer algún error o equivocación*<br>'to make some slip or mistake'

					'Today we don't llucer. Yesterday we llucerred a lot and Carlos also ….'	
-er	PRET	nonrhyme	1p	*naler*	*Hoy Rafaela no nale. Ayer ella nalió mucho y nosotros también ….*<br>'Today Rafaela doesn't naler. Yesterday she nalerred a lot and we also ....'	*mostrar gratitud por un favor*<br>'to show gratitude for a favor'
-er	PRET	nonrhyme	2p	*jaser*	*Hoy yo no jaso. Ayer jasí mucho y vosotros también ….*<br>'Today I don't jaser. Yesterday I jaserred a lot and you (pl) also ....'	*apagar fuego*<br>'to put out fire'
