# Supplementary material for: Literacy-related differences in morphological knowledge: A nonce-word study
Source: Front Psychol. 2023 Apr 26;14:1136337. doi: 10.3389/fpsyg.2023.1136337 (PMC10171427; doi:10.3389/fpsyg.2023.1136337)
Supplement: Supplementary file 3 [file Data_Sheet_2.pdf]

```
library(readxl)
library(lme4)
library(lmerTest)
library(MuMIn)

###Read dataframe
nonverbs <- read_excel("YOUR_PATH/nonce_dataset_Feb2022.xlsx")
summary(nonverbs)

####### Contrast coding procedure for Group
###HighVsRest contrasts the high literate group with the other two
groups: high vs late and semi
nonverbs$HighVsRest = rep(0,nrow(nonverbs))
nonverbs$HighVsRest[nonverbs$group=="high"]=0.5
nonverbs$HighVsRest[nonverbs$group=="late"]=-0.25
nonverbs$HighVsRest[nonverbs$group=="semi"]=-0.25

###LateVsSemi contrasts the late literate group with the semi-
literate group: late vs semi
nonverbs$LateVsSemi = rep(0,nrow(nonverbs))
nonverbs$LateVsSemi[nonverbs$group=="high"]=0
nonverbs$LateVsSemi[nonverbs$group=="semi"]=-0.5
nonverbs$LateVsSemi[nonverbs$group=="late"]=0.5

####Contrast-coding for PersonNumber (1P 2P 3S)
### P3SvsRest contasts 3S with the other values
nonverbs$PersNum = as.factor(nonverbs$PersNum)
nonverbs$P3SvsRest = rep(0,nrow(nonverbs))
nonverbs$P3SvsRest[nonverbs$PersNum=="3S"]=0.5
nonverbs$P3SvsRest[nonverbs$PersNum=="2P"]=-0.25
nonverbs$P3SvsRest[nonverbs$PersNum=="1P"]=-0.25

### P1PvsP2P contrasts 1st and 2nd person plural
nonverbs$P1PvsP2P = rep(0,nrow(nonverbs))
nonverbs$P1PvsP2P[nonverbs$PersNum=="3S"]=0
nonverbs$P1PvsP2P[nonverbs$PersNum=="2P"]=-0.5
nonverbs$P1PvsP2P[nonverbs$PersNum=="1P"]=0.5

###Contrast-coding for conjugation
nonverbs$ArVsEr = ifelse(nonverbs$ArEr == "AR", 0.5, -0.5)

###Contrast-coding for aspect
nonverbs$PretVsImp = ifelse(nonverbs$PretImp == "PRET", 0.5, -0.5)

#######################################################
### Regression analysis
#######################################################

###Base model with random intercepts for participant and verb only
###Note: strict means 1-0 coding based on the criteria specified in
the manuscript (conjugation, person, number, tense and aspect all
correct)
BigModel = glmer(strict   ~  HighVsRest + LateVsSemi + ArVsEr +
P3SvsRest + P1PvsP2P + PretVsImp + scale(Age) + scale(CPM) +
```

```
          HighVsRest:ArVsEr + LateVsSemi:ArVsEr +
HighVsRest:P3SvsRest + LateVsSemi:P3SvsRest + HighVsRest:P1PvsP2P +
LateVsSemi:P1PvsP2P + HighVsRest:PretVsImp + LateVsSemi:PretVsImp +
          (1|participant) + (1|Verb), data = nonverbs, family =
"binomial", control=glmerControl(optimizer='bobyqa'))
summary(BigModel)
#
# Generalized linear mixed model fit by maximum likelihood (Laplace
Approximation) ['glmerMod']
# Family: binomial  ( logit )
# Formula: strict ~ HighVsRest + LateVsSemi + ArVsEr + P3SvsRest +
P1PvsP2P +
#   PretVsImp + scale(Age) + scale(CPM) + HighVsRest:ArVsEr +
#   LateVsSemi:ArVsEr + HighVsRest:P3SvsRest + LateVsSemi:P3SvsRest
+
#   HighVsRest:P1PvsP2P + LateVsSemi:P1PvsP2P + HighVsRest:PretVsImp
+
#   LateVsSemi:PretVsImp + (1 | participant) + (1 | Verb)
# Data: nonverbs
# Control: glmerControl(optimizer = "bobyqa")
#
# AIC      BIC   logLik deviance df.resid
# 1141.0   1236.5   -551.5   1103.0     1109
#
# Scaled residuals:
#   Min      1Q  Median      3Q     Max
# -2.1608 -0.5240 -0.2602  0.6469 10.1114
#
# Random effects:
#   Groups      Name        Variance Std.Dev.
# participant (Intercept) 0.8694   0.9324
# Verb        (Intercept) 0.1441   0.3796
# Number of obs: 1128, groups:  participant, 47; Verb, 24
#
# Fixed effects:
#   Estimate Std. Error z value Pr(>|z|)
# (Intercept)          -1.10800    0.18627  -5.948 2.71e-09 ***
#   HighVsRest            1.34333    0.65062   2.065  0.03895 *
#   LateVsSemi            0.91098    0.42058   2.166  0.03031 *
#   ArVsEr                1.31773    0.22656   5.816 6.02e-09 ***
#   P3SvsRest             1.22948    0.31404   3.915 9.04e-05 ***
#   P1PvsP2P              1.27019    0.29802   4.262 2.02e-05 ***
#   PretVsImp            -0.09693    0.22211  -0.436  0.66255
# scale(Age)            0.30747    0.16599   1.852  0.06399 .
# scale(CPM)            0.36298    0.23000   1.578  0.11453
# HighVsRest:ArVsEr    -1.27120    0.44176  -2.878  0.00401 **
#   LateVsSemi:ArVsEr    -0.74123    0.41004  -1.808  0.07065 .
# HighVsRest:P3SvsRest -1.99951    0.61245  -3.265  0.00110 **
#   LateVsSemi:P3SvsRest -0.61695    0.56429  -1.093  0.27425
# HighVsRest:P1PvsP2P  -3.03810    0.58447  -5.198 2.01e-07 ***
#   LateVsSemi:P1PvsP2P  -1.06852    0.60226  -1.774  0.07603 .
# HighVsRest:PretVsImp -0.40345    0.43304  -0.932  0.35150
# LateVsSemi:PretVsImp -0.48526    0.38845  -1.249  0.21158
# ---
```

```
#   Signif. codes:  0 ‘***’ 0.001 ‘**’ 0.01 ‘*’ 0.05 ‘.’ 0.1 ‘ ’ 1

###Pseudo-Rsquared of the model above (MuMin package)
r.squaredGLMM(BigModel)
# R2m       R2c
# theoretical 0.3521168 0.5046943
# delta       0.2972035 0.4259862

###Add random slopes one by one and see if they improve model fit

## Add random slope for ArVsEr over Participant
slArVsEr = glmer(strict   ~  HighVsRest + LateVsSemi + ArVsEr +
P3SvsRest + P1PvsP2P + PretVsImp + scale(Age) + scale(CPM) +
                          HighVsRest:ArVsEr + LateVsSemi:ArVsEr +
HighVsRest:P3SvsRest + LateVsSemi:P3SvsRest + HighVsRest:P1PvsP2P +
LateVsSemi:P1PvsP2P + HighVsRest:PretVsImp + LateVsSemi:PretVsImp +
                          (ArVsEr|participant) + (1|Verb), data =
nonverbs, family = "binomial",
control=glmerControl(optimizer='bobyqa'))
anova(BigModel, slArVsEr)
## borderline significant (p = 0.0501), so we keep it

# Data: nonverbs
# Models:
#   BigModel: strict ~ HighVsRest + LateVsSemi + ArVsEr + P3SvsRest
+ P1PvsP2P + PretVsImp + scale(Age) + scale(CPM) + HighVsRest:ArVsEr
+ LateVsSemi:ArVsEr + HighVsRest:P3SvsRest + LateVsSemi:P3SvsRest +
HighVsRest:P1PvsP2P + LateVsSemi:P1PvsP2P + HighVsRest:PretVsImp +
LateVsSemi:PretVsImp + (1 | participant) + (1 | Verb)
# slArVsEr: strict ~ HighVsRest + LateVsSemi + ArVsEr + P3SvsRest +
P1PvsP2P + PretVsImp + scale(Age) + scale(CPM) + HighVsRest:ArVsEr +
LateVsSemi:ArVsEr + HighVsRest:P3SvsRest + LateVsSemi:P3SvsRest +
HighVsRest:P1PvsP2P + LateVsSemi:P1PvsP2P + HighVsRest:PretVsImp +
LateVsSemi:PretVsImp + (ArVsEr | participant) + (1 | Verb)
# npar  AIC    BIC  logLik deviance  Chisq Df Pr(>Chisq)
# BigModel   19 1141 1236.5 -551.50     1103
# slArVsEr   21 1139 1244.6 -548.51     1097 5.9911  2    0.05001 .
# ---
#   Signif. codes:  0 ‘***’ 0.001 ‘**’ 0.01 ‘*’ 0.05 ‘.’ 0.1 ‘ ’ 1


## Add random slope for P3SvsRest over Participant
slP3SvsRest = glmer(strict   ~  HighVsRest + LateVsSemi + ArVsEr +
P3SvsRest + P1PvsP2P + PretVsImp + scale(Age) + scale(CPM) +
                               HighVsRest:ArVsEr + LateVsSemi:ArVsEr
+ HighVsRest:P3SvsRest + LateVsSemi:P3SvsRest + HighVsRest:P1PvsP2P
+ LateVsSemi:P1PvsP2P + HighVsRest:PretVsImp + LateVsSemi:PretVsImp
+
                               (P3SvsRest|participant) + (1|Verb),
data = nonverbs, family = "binomial",
control=glmerControl(optimizer='bobyqa'))
anova(BigModel, slP3SvsRest)
## Doesn't improve model fit, so we drop it
```

```
# Data: nonverbs
# Models:
#   BigModel: strict ~ HighVsRest + LateVsSemi + ArVsEr + P3SvsRest
+ P1PvsP2P + PretVsImp + scale(Age) + scale(CPM) + HighVsRest:ArVsEr
+ LateVsSemi:ArVsEr + HighVsRest:P3SvsRest + LateVsSemi:P3SvsRest +
HighVsRest:P1PvsP2P + LateVsSemi:P1PvsP2P + HighVsRest:PretVsImp +
LateVsSemi:PretVsImp + (1 | participant) + (1 | Verb)
# slP3SvsRest: strict ~ HighVsRest + LateVsSemi + ArVsEr + P3SvsRest
+ P1PvsP2P + PretVsImp + scale(Age) + scale(CPM) + HighVsRest:ArVsEr
+ LateVsSemi:ArVsEr + HighVsRest:P3SvsRest + LateVsSemi:P3SvsRest +
HighVsRest:P1PvsP2P + LateVsSemi:P1PvsP2P + HighVsRest:PretVsImp +
LateVsSemi:PretVsImp + (P3SvsRest | participant) + (1 | Verb)
# npar    AIC    BIC  logLik deviance Chisq Df Pr(>Chisq)
# BigModel      19 1141.0 1236.5 -551.50   1103.0
# slP3SvsRest   21 1144.9 1250.5 -551.47   1102.9 0.064  2
0.9685


## Add random slope for P1PvsP2P over Participant
slP1PvsP2P = glmer(strict   ~  HighVsRest + LateVsSemi + ArVsEr +
P3SvsRest + P1PvsP2P + PretVsImp + scale(Age) + scale(CPM) +
                              HighVsRest:ArVsEr + LateVsSemi:ArVsEr
+ HighVsRest:P3SvsRest + LateVsSemi:P3SvsRest + HighVsRest:P1PvsP2P
+ LateVsSemi:P1PvsP2P + HighVsRest:PretVsImp + LateVsSemi:PretVsImp
+
                              (P1PvsP2P|participant) + (1|Verb),
data = nonverbs, family = "binomial",
control=glmerControl(optimizer='bobyqa'))
anova(BigModel, slP1PvsP2P)
## Marginally significant (p=0.079). We keep it too.

# Data: nonverbs
# Models:
#   BigModel: strict ~ HighVsRest + LateVsSemi + ArVsEr + P3SvsRest
+ P1PvsP2P + PretVsImp + scale(Age) + scale(CPM) + HighVsRest:ArVsEr
+ LateVsSemi:ArVsEr + HighVsRest:P3SvsRest + LateVsSemi:P3SvsRest +
HighVsRest:P1PvsP2P + LateVsSemi:P1PvsP2P + HighVsRest:PretVsImp +
LateVsSemi:PretVsImp + (1 | participant) + (1 | Verb)
# slP1PvsP2P: strict ~ HighVsRest + LateVsSemi + ArVsEr + P3SvsRest
+ P1PvsP2P + PretVsImp + scale(Age) + scale(CPM) + HighVsRest:ArVsEr
+ LateVsSemi:ArVsEr + HighVsRest:P3SvsRest + LateVsSemi:P3SvsRest +
HighVsRest:P1PvsP2P + LateVsSemi:P1PvsP2P + HighVsRest:PretVsImp +
LateVsSemi:PretVsImp + (P1PvsP2P | participant) + (1 | Verb)
# npar    AIC    BIC  logLik deviance  Chisq Df Pr(>Chisq)
# BigModel     19 1141.0 1236.5 -551.50   1103.0
# slP1PvsP2P   21 1139.9 1245.5 -548.96   1097.9 5.0826  2
0.07876 .
# ---
#   Signif. codes:  0 ‘***’ 0.001 ‘**’ 0.01 ‘*’ 0.05 ‘.’ 0.1 ‘ ’ 1

## Add random slope for PretVsImp over Participant
slPretVsImp = glmer(strict   ~  HighVsRest + LateVsSemi + ArVsEr +
P3SvsRest + P1PvsP2P + PretVsImp + scale(Age) + scale(CPM) +
                           HighVsRest:ArVsEr + LateVsSemi:ArVsEr +
```

```
HighVsRest:P3SvsRest + LateVsSemi:P3SvsRest + HighVsRest:P1PvsP2P +
LateVsSemi:P1PvsP2P + HighVsRest:PretVsImp + LateVsSemi:PretVsImp +
                           (PretVsImp|participant) + (1|Verb), data
= nonverbs, family = "binomial",
control=glmerControl(optimizer='bobyqa'))
anova(BigModel, slPretVsImp)
## Improves model fit so we keep it

# Data: nonverbs
# Models:
#   BigModel: strict ~ HighVsRest + LateVsSemi + ArVsEr + P3SvsRest
+ P1PvsP2P + PretVsImp + scale(Age) + scale(CPM) + HighVsRest:ArVsEr
+ LateVsSemi:ArVsEr + HighVsRest:P3SvsRest + LateVsSemi:P3SvsRest +
HighVsRest:P1PvsP2P + LateVsSemi:P1PvsP2P + HighVsRest:PretVsImp +
LateVsSemi:PretVsImp + (1 | participant) + (1 | Verb)
# slPretVsImp: strict ~ HighVsRest + LateVsSemi + ArVsEr + P3SvsRest
+ P1PvsP2P + PretVsImp + scale(Age) + scale(CPM) + HighVsRest:ArVsEr
+ LateVsSemi:ArVsEr + HighVsRest:P3SvsRest + LateVsSemi:P3SvsRest +
HighVsRest:P1PvsP2P + LateVsSemi:P1PvsP2P + HighVsRest:PretVsImp +
LateVsSemi:PretVsImp + (PretVsImp | participant) + (1 | Verb)
# npar  AIC    BIC  logLik deviance  Chisq Df Pr(>Chisq)
# BigModel      19 1141 1236.5 -551.50     1103
# slPretVsImp   21 1131 1236.6 -544.49     1089 14.018  2  0.0009037
***
#   ---
#   Signif. codes:  0 ‘***’ 0.001 ‘**’ 0.01 ‘*’ 0.05 ‘.’ 0.1 ‘ ’ 1

## Add random slope for HighVsRest over Verb
slHighVsRest = glmer(strict   ~  HighVsRest + LateVsSemi + ArVsEr +
P3SvsRest + P1PvsP2P + PretVsImp + scale(Age) + scale(CPM) +
                            HighVsRest:ArVsEr + LateVsSemi:ArVsEr +
HighVsRest:P3SvsRest + LateVsSemi:P3SvsRest + HighVsRest:P1PvsP2P +
LateVsSemi:P1PvsP2P + HighVsRest:PretVsImp + LateVsSemi:PretVsImp +
                            (1|participant) + (HighVsRest|Verb),
data = nonverbs, family = "binomial",
control=glmerControl(optimizer='bobyqa'))
anova(BigModel, slHighVsRest)
## Doesn't improve model fit, so we drop it

# Data: nonverbs
# Models:
#   BigModel: strict ~ HighVsRest + LateVsSemi + ArVsEr + P3SvsRest
+ P1PvsP2P + PretVsImp + scale(Age) + scale(CPM) + HighVsRest:ArVsEr
+ LateVsSemi:ArVsEr + HighVsRest:P3SvsRest + LateVsSemi:P3SvsRest +
HighVsRest:P1PvsP2P + LateVsSemi:P1PvsP2P + HighVsRest:PretVsImp +
LateVsSemi:PretVsImp + (1 | participant) + (1 | Verb)
# slHighVsRest: strict ~ HighVsRest + LateVsSemi + ArVsEr +
P3SvsRest + P1PvsP2P + PretVsImp + scale(Age) + scale(CPM) +
HighVsRest:ArVsEr + LateVsSemi:ArVsEr + HighVsRest:P3SvsRest +
LateVsSemi:P3SvsRest + HighVsRest:P1PvsP2P + LateVsSemi:P1PvsP2P +
HighVsRest:PretVsImp + LateVsSemi:PretVsImp + (1 | participant) +
(HighVsRest | Verb)
# npar  AIC    BIC  logLik deviance Chisq Df Pr(>Chisq)
# BigModel       19 1141 1236.5 -551.50     1103
```

```
# slHighVsRest   21 1143 1248.6 -550.48     1101 2.034  2     0.3617

## Add random slope for LateVsSemi over Verb
slLateVsSemi = glmer(strict   ~  HighVsRest + LateVsSemi + ArVsEr +
P3SvsRest + P1PvsP2P + PretVsImp + scale(Age) + scale(CPM) +
                            HighVsRest:ArVsEr + LateVsSemi:ArVsEr +
HighVsRest:P3SvsRest + LateVsSemi:P3SvsRest + HighVsRest:P1PvsP2P +
LateVsSemi:P1PvsP2P + HighVsRest:PretVsImp + LateVsSemi:PretVsImp +
                            (1|participant) + (LateVsSemi|Verb),
data = nonverbs, family = "binomial",
control=glmerControl(optimizer='bobyqa'))
anova(BigModel, slLateVsSemi)
## Doesn't improve model fit, so we drop it

# Data: nonverbs
# Models:
#   BigModel: strict ~ HighVsRest + LateVsSemi + ArVsEr + P3SvsRest
+ P1PvsP2P + PretVsImp + scale(Age) + scale(CPM) + HighVsRest:ArVsEr
+ LateVsSemi:ArVsEr + HighVsRest:P3SvsRest + LateVsSemi:P3SvsRest +
HighVsRest:P1PvsP2P + LateVsSemi:P1PvsP2P + HighVsRest:PretVsImp +
LateVsSemi:PretVsImp + (1 | participant) + (1 | Verb)
# slLateVsSemi: strict ~ HighVsRest + LateVsSemi + ArVsEr +
P3SvsRest + P1PvsP2P + PretVsImp + scale(Age) + scale(CPM) +
HighVsRest:ArVsEr + LateVsSemi:ArVsEr + HighVsRest:P3SvsRest +
LateVsSemi:P3SvsRest + HighVsRest:P1PvsP2P + LateVsSemi:P1PvsP2P +
HighVsRest:PretVsImp + LateVsSemi:PretVsImp + (1 | participant) +
(LateVsSemi | Verb)
# npar  AIC    BIC  logLik deviance  Chisq Df Pr(>Chisq)
# BigModel       19 1141 1236.5 -551.50     1103
# slLateVsSemi   21 1144 1249.5 -550.98     1102 1.0472  2
0.5924

#### Final model: random slopes for ArVsEr, P1PvsP2P and PretVsImp
over participants added to initial model
FinalModel = glmer(strict   ~  HighVsRest + LateVsSemi + ArVsEr +
P3SvsRest + P1PvsP2P + PretVsImp + scale(Age) + scale(CPM) +
                        HighVsRest:ArVsEr + LateVsSemi:ArVsEr +
HighVsRest:P3SvsRest + LateVsSemi:P3SvsRest + HighVsRest:P1PvsP2P +
LateVsSemi:P1PvsP2P + HighVsRest:PretVsImp + LateVsSemi:PretVsImp +
                        (ArVsEr + P1PvsP2P +PretVsImp|participant) +
(1|Verb), data = nonverbs, family = "binomial",
control=glmerControl(optimizer='bobyqa'))
summary(FinalModel)

# Generalized linear mixed model fit by maximum likelihood (Laplace
Approximation) ['glmerMod']
# Family: binomial  ( logit )
# Formula: strict ~ HighVsRest + LateVsSemi + ArVsEr + P3SvsRest +
P1PvsP2P +
#   PretVsImp + scale(Age) + scale(CPM) + HighVsRest:ArVsEr +
#   LateVsSemi:ArVsEr + HighVsRest:P3SvsRest + LateVsSemi:P3SvsRest
+
#   HighVsRest:P1PvsP2P + LateVsSemi:P1PvsP2P + HighVsRest:PretVsImp
+
```

```
#   LateVsSemi:PretVsImp + (ArVsEr + P1PvsP2P + PretVsImp |
participant) +      (1 | Verb)
# Data: nonverbs
# Control: glmerControl(optimizer = "bobyqa")
#
# AIC      BIC   logLik deviance df.resid
# 1129.1   1269.9   -536.5   1073.1     1100
#
# Scaled residuals:
#   Min      1Q  Median      3Q     Max
# -2.4266 -0.4779 -0.2038  0.5648  9.2611
#
# Random effects:
#   Groups      Name        Variance Std.Dev. Corr
# participant (Intercept) 1.3698   1.1704
# ArVsEr      0.8364   0.9145   -0.16
# P1PvsP2P    0.8535   0.9239   -0.85 -0.38
# PretVsImp   1.4436   1.2015   -0.26 -0.21  0.28
# Verb        (Intercept) 0.1962   0.4430
# Number of obs: 1128, groups:  participant, 47; Verb, 24
#
# Fixed effects:
#   Estimate Std. Error z value Pr(>|z|)
# (Intercept)          -1.29369    0.23139  -5.591 2.26e-08 ***
#   HighVsRest            2.04026    0.75851   2.690 0.007149 **
#   LateVsSemi            1.14131    0.51342   2.223 0.026217 *
#   ArVsEr                1.50269    0.30268   4.965 6.88e-07 ***
#   P3SvsRest             1.45403    0.35958   4.044 5.26e-05 ***
#   P1PvsP2P              1.60162    0.38457   4.165 3.12e-05 ***
#   PretVsImp            -0.09912    0.31641  -0.313 0.754082
# scale(Age)            0.48056    0.18426   2.608 0.009107 **
#   scale(CPM)            0.19994    0.23972   0.834 0.404235
# HighVsRest:ArVsEr    -1.37002    0.62840  -2.180 0.029244 *
#   LateVsSemi:ArVsEr    -0.95407    0.56539  -1.687 0.091516 .
# HighVsRest:P3SvsRest -2.37550    0.67328  -3.528 0.000418 ***
#   LateVsSemi:P3SvsRest -0.73915    0.61979  -1.193 0.233031
# HighVsRest:P1PvsP2P  -3.53071    0.77294  -4.568 4.93e-06 ***
#   LateVsSemi:P1PvsP2P  -1.36110    0.76185  -1.787 0.074004 .
# HighVsRest:PretVsImp -0.44274    0.70530  -0.628 0.530184
# LateVsSemi:PretVsImp -0.63185    0.60695  -1.041 0.297862
# ---
#   Signif. codes:  0 ‘***’ 0.001 ‘**’ 0.01 ‘*’ 0.05 ‘.’ 0.1 ‘ ’ 1


###Pseudo-Rsquared of the final model
r.squaredGLMM(FinalModel)
# R2m       R2c
# theoretical 0.3727376 0.6293867
# delta       0.3328091 0.5619654


##########################################
```

```
### Follow-up analysis: Effect of IQ
##########################################

###Base model: group variables, IQ and interactions between group
and IQ
IQ_followup = glmer(strict   ~  HighVsRest + LateVsSemi + scale(CPM)
+ HighVsRest:scale(CPM) + LateVsSemi:scale(CPM) + (1|participant) +
(1|Verb), data = nonverbs, family = "binomial",
control=glmerControl(optimizer='bobyqa'))
summary(IQ_followup)

## Add random slope for HighVsRest over Verb
IQ_followup1 = glmer(strict   ~  HighVsRest + LateVsSemi +
scale(CPM) + HighVsRest:scale(CPM) + LateVsSemi:scale(CPM) + (1|
participant) + (HighVsRest|Verb), data = nonverbs, family =
"binomial", control=glmerControl(optimizer='bobyqa'))
anova(IQ_followup,IQ_followup1)
## Improves model fit so we keep it

# Data: nonverbs
# Models:
#   IQ_followup: strict ~ HighVsRest + LateVsSemi + scale(CPM) +
HighVsRest:scale(CPM) +
#   IQ_followup:     LateVsSemi:scale(CPM) + (1 | participant) + (1
| Verb)
# IQ_followup1: strict ~ HighVsRest + LateVsSemi + scale(CPM) +
HighVsRest:scale(CPM) +
#   IQ_followup1:     LateVsSemi:scale(CPM) + (1 | participant) +
(HighVsRest |
#
IQ_followup1:     Verb)
# npar    AIC    BIC  logLik deviance  Chisq Df Pr(>Chisq)
# IQ_followup     8 1196.7 1237.0 -590.37   1180.7
# IQ_followup1   10 1172.0 1222.2 -575.98   1152.0 28.773  2
5.65e-07 ***
#   ---
#   Signif. codes:  0 ‘***’ 0.001 ‘**’ 0.01 ‘*’ 0.05 ‘.’ 0.1 ‘ ’ 1

IQ_followup2 = glmer(strict   ~  HighVsRest + LateVsSemi +
scale(CPM) + HighVsRest:scale(CPM) + LateVsSemi:scale(CPM) + (1|
participant) + (LateVsSemi|Verb), data = nonverbs, family =
"binomial", control=glmerControl(optimizer='bobyqa'))
summary(IQ_followup2)
anova(IQ_followup,IQ_followup2)
## Improves model fit but results in singular fit; we keep
IQ_followup1 as best model

summary(IQ_followup1)
# Generalized linear mixed model fit by maximum likelihood (Laplace
Approximation) ['glmerMod']
# Family: binomial  ( logit )
# Formula: strict ~ HighVsRest + LateVsSemi + scale(CPM) +
HighVsRest:scale(CPM) +
#   LateVsSemi:scale(CPM) + (1 | participant) + (HighVsRest |
```

```
Verb)
# Data: nonverbs
# Control: glmerControl(optimizer = "bobyqa")
#
# AIC      BIC   logLik deviance df.resid
# 1172.0   1222.2   -576.0   1152.0     1118
#
# Scaled residuals:
#   Min      1Q  Median      3Q     Max
# -2.0875 -0.5230 -0.2806  0.6089  3.8510
#
# Random effects:
#   Groups      Name        Variance Std.Dev. Corr
# participant (Intercept) 0.924    0.9613
# Verb        (Intercept) 1.005    1.0023
# HighVsRest  2.260    1.5032   -0.88
# Number of obs: 1128, groups:  participant, 47; Verb, 24
#
# Fixed effects:
#   Estimate Std. Error z value Pr(>|z|)
# (Intercept)            -1.3136     0.4116  -3.192  0.00142 **
#   HighVsRest              0.3007     1.3586   0.221  0.82486
# LateVsSemi             0.8107     0.4451   1.821  0.06856 .
# scale(CPM)             0.5044     0.3253   1.551  0.12101
# HighVsRest:scale(CPM)   0.9575     1.1725   0.817  0.41415
# LateVsSemi:scale(CPM)   0.1242     0.4871   0.255  0.79873
# ---
#   Signif. codes:  0 ‘***’ 0.001 ‘**’ 0.01 ‘*’ 0.05 ‘.’ 0.1 ‘ ’ 1
#
# Correlation of Fixed Effects:
#   (Intr) HghVsR LtVsSm s(CPM) HVR:(C
#                                   HighVsRest   0.560
#                                   LateVsSemi   0.000 -0.002
#                                   scale(CPM)  -0.572 -0.830
-0.052
#                                   HghVR:(CPM) -0.760 -0.844
0.030  0.694
#                                   LtVsS:(CPM) -0.040  0.024
0.428  0.070 -0.039


#####Additional analysis with IQ as dependent variable and group as
independent variable (one value per participant -> multiple
regression)

IQ_agg = with(nonverbs, aggregate(CPM,
list(participant,group,HighVsRest,LateVsSemi), mean,na.rm = TRUE))
colnames(IQ_agg) <-
c("participant","group","HighVsRest","LateVsSemi","CPM")


### Mean and SD CPM by group
with(IQ_agg, tapply(CPM, list(group), mean))
```

```
# high     late     semi
# 30.64286 21.84615 19.80000

with(IQ_agg, tapply(CPM, list(group), sd))

# high     late     semi
# 2.307418 5.550468 5.217380


###Multiple regression model: CPM as dependent variable; HighVsRest
and LateVsSemi as predictors
reg_IQ = lm(CPM ~ HighVsRest + LateVsSemi, data = IQ_agg)
summary(reg_IQ)

# Call:
#   lm(formula = CPM ~ HighVsRest + LateVsSemi, data = IQ_agg)
#
# Coefficients:
#   (Intercept)   HighVsRest   LateVsSemi
# 24.096       13.093        2.046
#
# > reg_IQ = lm(CPM ~ HighVsRest + LateVsSemi, data = IQ_agg)
# > summary(reg_IQ)
#
# Call:
#   lm(formula = CPM ~ HighVsRest + LateVsSemi, data = IQ_agg)
#
# Residuals:
#   Min     1Q Median     3Q    Max
# -7.846 -3.221  0.200  2.357 11.200
#
# Coefficients:
#   Estimate Std. Error t value Pr(>|t|)
# (Intercept)   24.096      0.692  34.820  < 2e-16 ***
#   HighVsRest    13.093      1.996   6.559 5.08e-08 ***
#   LateVsSemi     2.046      1.661   1.232    0.224
# ---
#   Signif. codes:  0 ‘***’ 0.001 ‘**’ 0.01 ‘*’ 0.05 ‘.’ 0.1 ‘ ’ 1
#
# Residual standard error: 4.662 on 44 degrees of freedom
# Multiple R-squared:  0.517,   Adjusted R-squared:  0.495
# F-statistic: 23.54 on 2 and 44 DF,  p-value: 1.116e-07





##########################################
### Descriptives (Tables 4 and 5)
##########################################

###Aggregate by participant for each of the variables of interest
nonce = with(nonverbs, aggregate(Nonce, list(participant,group),
mean,na.rm = TRUE))
```

```
stem = with(nonverbs, aggregate(Stem, list(participant,group),
mean,na.rm = TRUE))
person = with(nonverbs, aggregate(Person, list(participant,group),
mean,na.rm = TRUE))
number = with(nonverbs, aggregate(Number, list(participant,group),
mean,na.rm = TRUE))
conj = with(nonverbs, aggregate(Conj, list(participant,group),
mean,na.rm = TRUE))
tense = with(nonverbs, aggregate(Tense, list(participant,group),
mean,na.rm = TRUE))
aspect = with(nonverbs, aggregate(Aspect, list(participant,group),
mean,na.rm = TRUE))
CPM = with(nonverbs, aggregate(CPM, list(participant,group),
mean,na.rm = TRUE))
strict = with(nonverbs, aggregate(strict, list(participant,group),
mean,na.rm = TRUE))

###Change column names consistently
colnames(nonce) <- c("participant","group","nonce")
colnames(stem) <- c("participant","group","stem")
colnames(person) <- c("participant","group","person")
colnames(number) <- c("participant","group","number")
colnames(conj) <- c("participant","group","conj")
colnames(tense) <- c("participant","group","tense")
colnames(aspect) <- c("participant","group","aspect")
colnames(CPM) <- c("participant","group","CPM")
colnames(strict) <- c("participant","group","strict")

###Table 4

### Mean and SD nonce verb status
with(nonce, tapply(nonce, list(group), mean, na.rm=TRUE))
# high      late      semi
# 0.9940476 0.9839744 0.9560688
with(nonce, tapply(nonce, list(group), sd, na.rm=TRUE))
# high       late       semi
# 0.01513069 0.04670404 0.03717255


### Mean and SD correct stem
with(stem, tapply(stem, list(group), mean, na.rm=TRUE))
# high      late      semi
# 0.9462992 0.7853324 0.6012705

with(stem, tapply(stem, list(group), sd, na.rm=TRUE))
# high       late       semi
# 0.07384623 0.14015520 0.14509106


### Mean and SD person
with(person, tapply(person, list(group), mean, na.rm=TRUE))
# high      late      semi
# 0.9846014 0.8077633 0.6549760
```

```
with(person, tapply(person, list(group), sd, na.rm=TRUE))
# high       late       semi
# 0.04695517 0.14854540 0.15834713


### Mean and SD number
with(number, tapply(number, list(group), mean, na.rm=TRUE))
# high      late      semi
# 1.0000000 0.9418238 0.8566123

with(number, tapply(number, list(group), sd, na.rm=TRUE))
# high       late       semi
# 0.00000000 0.08075557 0.15437706


### Mean and SD conjugation
with(conj, tapply(conj, list(group), mean, na.rm=TRUE))
# high      late      semi
# 0.8117236 0.7095242 0.6832698

with(conj, tapply(conj, list(group), sd, na.rm=TRUE))
# high      late      semi
# 0.1113991 0.1013770 0.1217532


### Mean and SD tense
with(tense, tapply(tense, list(group), mean, na.rm=TRUE))
# high      late      semi
# 0.7500000 0.7909902 0.7369095

with(tense, tapply(tense, list(group), sd, na.rm=TRUE))
# high      late      semi
# 0.3313241 0.1719756 0.2582407


### Mean and SD aspect
with(aspect, tapply(aspect, list(group), mean, na.rm=TRUE))
# high      late      semi
# 0.8035037 0.7169816 0.6766949

with(aspect, tapply(aspect, list(group), sd, na.rm=TRUE))
# high      late      semi
# 0.1926188 0.1685340 0.1237375


### Mean and SD IQ (CPM scores)
with(CPM, tapply(CPM, list(group), mean, na.rm=TRUE))
# high     late     semi
# 30.64286 21.84615 19.80000

with(CPM, tapply(CPM, list(group), sd, na.rm=TRUE))
# high     late     semi
# 2.307418 5.550468 5.217380
```

```
### Mean and SD strict coding criterion: used for analyses above
with(strict, tapply(strict, list(group), mean, na.rm=TRUE))
# high      late      semi
# 0.4970238 0.3237179 0.2104167

with(strict, tapply(strict, list(group), sd, na.rm=TRUE))
# high      late      semi
# 0.2921079 0.1180398 0.1051272



###Create data frame to get values for Table 5
detailed = with(nonverbs, aggregate(strict,
list(participant,group,ArEr,PersNum,PretImp), mean,na.rm = TRUE))
colnames(detailed) <-
c("participant","group","conjugation","person","aspect","correct")

### Mean and SD for all possible combinations of group, conjugation,
person and aspect
with(detailed, tapply(correct,
list(group,conjugation,person,aspect), mean,na.rm = TRUE))

#         , , 1P, IMP
#
#           AR        ER
# high 0.6428571 0.5000000
# late 0.5000000 0.2307692
# semi 0.3000000 0.0750000
#
# , , 2P, IMP
#
#           AR        ER
# high 0.5714286 0.5357143
# late 0.3076923 0.1153846
# semi 0.1250000 0.0250000
#
# , , 3S, IMP
#
#           AR        ER
# high 0.6428571 0.2500000
# late 0.5769231 0.3076923
# semi 0.5500000 0.0750000
#
# , , 1P, PRET
#
#         AR        ER
# high 0.4642857 0.2500000
# late 0.6923077 0.1153846
# semi 0.6500000 0.1000000
#
# , , 2P, PRET
#
#         AR         ER
```

```
# high 0.4285714 0.50000000
# late 0.1153846 0.03846154
# semi 0.0000000 0.00000000
#
# , , 3S, PRET
#
#          AR        ER
# high 0.6071429 0.5714286
# late 0.4230769 0.4615385
# semi 0.3750000 0.2500000


with(detailed, tapply(correct,
list(group,conjugation,person,aspect), sd,na.rm = TRUE))

# , , 1P, IMP
#
#          AR        ER
# high 0.4569368 0.4385290
# late 0.5000000 0.3881250
# semi 0.4412900 0.1831738
#
# , , 2P, IMP
#
# AR        ER
# high 0.4746311 0.4986245
# late 0.3839738 0.2995723
# semi 0.3193332 0.1118034
#
# , , 3S, IMP
#
#          AR        ER
# high 0.4127102 0.3252218
# late 0.3443686 0.3839738
# semi 0.4261208 0.2446802
#
# , , 1P, PRET
#
#            AR        ER
# high 0.4143710 0.3797773
# late 0.3839738 0.2192645
# semi 0.4322524 0.2615742
#
# , , 2P, PRET
#
#          AR       ER
# high 0.4322189 0.438529
# late 0.2995723 0.138675
# semi 0.0000000 0.000000
#
# , , 3S, PRET
#
# AR        ER
# high 0.4462912 0.4746311
```

```
# late 0.4003204 0.4311582
# semi 0.4253482 0.3441236
#



##########################################
### Figures 2 and 3
##########################################

##############Figure 2
####Create data frame with individual scores by group by conjugation
GroupConj = with(nonverbs, aggregate(strict,
list(participant,group,ArEr), mean,na.rm = TRUE))
colnames(GroupConj) <-
c("participant","group","conjugation","correct")

with(GroupConj, tapply(correct, list(group,conjugation), mean,na.rm
= TRUE))
####These are the means plotted in Figure 2 (see below)

# AR        ER
# high 0.5595238 0.4345238
# late 0.4358974 0.2115385
# semi 0.3333333 0.0875000


##### Get means and SD per group per conjugation
GCmeans = with(GroupConj, tapply(correct*100,
list(conjugation,group), mean))
GCsd = with(GroupConj, tapply(correct*100, list(conjugation,group),
sd))

#### Get standard error for sample size
GCSE = GCsd/sqrt(47)

#### Upper and lower limits
GCHW <- GCmeans+GCSE      # upper value
GCLW <- GCmeans-GCSE      # lower value


#####Figure 2 plot

barLocs <- barplot(GCmeans, space=c(0,0.5),ylim = c(0,100), beside =
T, col=gray(c(0.1,0.9), alpha = NULL),
                   xpd=FALSE,ylab = "",xlab="", legend =F, main =
"",cex.axis = 1.1,cex.lab=1.3,

cex=1.2,cex.main=1.95,names=c("high","late","semi"))
# bars
arrows(barLocs, GCLW, barLocs, GCHW, length = 0.1, lwd = 1, angle =
90, code = 3)     # error bars
title(ylab="Percentage correct responses", line=2.55, cex.lab=1.3,
family="")
```

```
legend(4, 95, cex = 1, bty = "n",
       legend = c("-AR verbs", "-ER verbs"),
       fill = gray(c(0.2,0.8)))
abline(h=0, col="black",lwd = 1.5)




###################Figure 3
####Create data frame with individual scores by group by person/verb
form
Group3sVsRest = with(nonverbs, aggregate(strict,
list(participant,group,PersNum), mean,na.rm = TRUE))
colnames(Group3sVsRest) <-
c("participant","group","person","correct")

with(Group3sVsRest, tapply(correct, list(group,person), mean,na.rm =
TRUE))
####These are the means plotted in Figure 3 (see below)

#         1P        2P        3S
# high 0.4642857 0.5089286 0.5178571
# late 0.3846154 0.1442308 0.4423077
# semi 0.2812500 0.0375000 0.3125000




###Change level names to get the desired order (3rd singular before
plural forms)
levels (Group3sVsRest$person) <- list(a3S= "3S", z1P= "1P", z2P =
"2P")
levels (Group3sVsRest$person)

##### Get means and SD per group per person
GPmeans = with(Group3sVsRest, tapply(correct*100,
list(person,group), mean))
GPsd = with(Group3sVsRest, tapply(correct*100, list(person,group),
sd))

#### Get standard error for sample size
GPSE = GPsd/sqrt(47)

#### Upper and lower limits
GPHW <- GPmeans+GPSE      # upper value
GPLW <- GPmeans-GPSE      # lower value


#####Figure 3 plot

barLocs2 <- barplot(GPmeans, space=c(0,0.5),ylim = c(0,100), beside
= T, col=gray(c(0.1,0.5,0.9), alpha = NULL),
                   xpd=FALSE,ylab = "",xlab="", legend =F, main =
```

```
"",cex.axis = 1.1,cex.lab=1.3,

cex=1.2,cex.main=1.95,names=c("high","late","semi"))
# bars
arrows(barLocs2, GPLW, barLocs2, GPHW, length = 0.1, lwd = 1, angle
= 90, code = 3)     # error bars
title(ylab="Percentage correct responses", line=2.55, cex.lab=1.3,
family="")
legend(6, 95, cex = 1, bty = "n",
       legend = c("3rd singular", "1st plural", "2nd plural"),
       fill = gray(c(0.2,0.5,0.8)))
abline(h=0, col="black",lwd = 1.5)
```
